# ADA2 regulates inflammation and hematopoietic stem cell emergence via the A_2b_R pathway in zebrafish

**DOI:** 10.1038/s42003-024-06286-3

**Published:** 2024-05-22

**Authors:** Alessia Brix, Laura Belleri, Alex Pezzotta, Emanuela Pettinato, Mara Mazzola, Matteo Zoccolillo, Anna Marozzi, Rui Monteiro, Filippo Del Bene, Alessandra Mortellaro, Anna Pistocchi

**Affiliations:** 1 Department of Medical Biotechnology and Translational Medicine, Università degli Studi di Milano, L.I.T.A., via Fratelli Cervi 93, Segrate, 20054, Milan, Italy; 2 Department of Development, Institut de la Vision, 17 Rue Moreau, 75012 Paris, France; 3 San Raffaele Telethon Institute for Gene Therapy (SR-Tiget), IRCCS San Raffaele Scientific Institute, via Olgettina 60, 20132 Milan, Italy; 4 Institute of Cancer and Genomic Sciences, University of Birmingham, Birmingham, Edgbaston, B15 2TTB UK

**Keywords:** Haematopoietic stem cells, Chronic inflammation

## Abstract

Deficiency of adenosine deaminase 2 (DADA2) is an inborn error of immunity caused by loss-of-function mutations in the adenosine deaminase 2 (*ADA2*) gene. Clinical manifestations of DADA2 include vasculopathy and immuno-hematological abnormalities, culminating in bone marrow failure. A major gap exists in our knowledge of the regulatory functions of ADA2 during inflammation and hematopoiesis, mainly due to the absence of an *ADA2* orthologue in rodents. Exploring these mechanisms is essential for understanding disease pathology and developing new treatments. Zebrafish possess two *ADA2* orthologues, *cecr1a* and *cecr1b*, with the latter showing functional conservation with human ADA2. We establish a *cecr1b*-loss-of-function zebrafish model that recapitulates the immuno-hematological and vascular manifestations observed in humans. Loss of Cecr1b disrupts hematopoietic stem cell specification, resulting in defective hematopoiesis. This defect is caused by induced inflammation in the vascular endothelium. Blocking inflammation, pharmacological modulation of the A_2_r pathway, or the administration of the recombinant human ADA2 corrects these defects, providing insights into the mechanistic link between ADA2 deficiency, inflammation and immuno-hematological abnormalities. Our findings open up potential therapeutic avenues for DADA2 patients.

## Abstract (web summary)

A DADA2 zebrafish model with *cecr1b* deficiency was generated. Characterization of hematopoietic processes revealed a role of Cecr1b in regulating inflammation and hematopoietic stem cell production through the A_2b_r-mediated adenosine signaling.

## Introduction

The deficiency of adenosine deaminase 2 (DADA2) is a rare genetic disorder due to recessive loss-of-function (LoF) mutations in the adenosine deaminase 2 (*ADA2*) gene (previously named *CECR1*)^1–3^. Patients affected by DADA2 present a broad spectrum of potentially lethal clinical manifestations, including immuno-hematological impairments [e.g., neutropenia and bone marrow aplasia], chronic systemic inflammation and endothelial cell dysfunction leading to intracranial leakage and stroke^4–8^. The precise biological function of ADA2 and the pathogenetic mechanisms causing such a wide range of phenotypes are unclear. As a result, current treatments alleviate hematological symptoms of DADA2, such as G-CSF, or reduce inflammation like anti-TNF agents, but they fail to offer a definitive cure^5,9–12^. Allogeneic hematopoietic stem cell transplantation stands as the only therapeutic option capable of completely and definitively correcting both the inflammatory and hematological phenotypes of DADA2 in patients^13–17^. This suggests that hematopoietic stem cells are the primary source of disease manifestations. However, the limited availability of compatible donors and the substantial risk of severe, life-threatening complications of allogeneic hematopoietic stem cell transplantation necessitate exploring alternative curative strategies^13,18^.

The lack of suitable animal models has posed a major obstacle to advancements in medical treatments for ADA2-related disorders, as rodents do not harbor ADA2 orthologues. Zebrafish possess two ADA2 orthologues, *cecr1a* and *cecr1b*, with *cecr1b* sharing a conserved function with human ADA2^1^. The LoF of *cecr1b*, but not *cecr1a*, leads to neutropenia and intracranial hemorrhages, successfully corrected by overexpressing human *ADA2* mRNA^1^. This compelling evidence, coupled with the remarkable conservation of molecular and cellular processes implicated in immuno-hematological and vascular homeostasis, positions zebrafish as a highly promising model for investigating the contribution of ADA2 in various aspects of DADA2 pathophysiology^19–23^.

ADA2, primarily expressed and secreted by myeloid cells, is a crucial enzyme involved in the catalytic pathway responsible for converting adenosine (Ado) into inosine^24,25^. The current limited understanding of ADA2 activity is derived from sequence homology studies with related enzymes such as ADA1 and adenosine deaminase growth factors^26,27^. ADA2 exhibits dual activities, acting as a growth factor by interacting with membrane proteoglycans and Ado receptors on immune cells and as an enzyme involved in Ado metabolism^26–31^. ADA2 is essential for the proper maturation of monocytes and the maintenance of macrophages in an anti-inflammatory M2 status, suggesting its important role in immune regulation^31–33^. Additionally, ADA2 enzymatic activity contributes to the Ado metabolism. High plasma ADA2 levels are associated with infections, autoimmunity, and chronic inflammation, potentially aiding in the degradation of excessive extracellular adenosine (eAdo) during inflammatory processes^30,34–39^. Recent findings suggest that the dysregulation of eAdo homeostasis may be involved in the pathogenesis of DADA2, as elevated levels of eAdo have been observed in the plasma of patients with DADA2^40^. In addition, patients with little or no ADA2 enzyme activity often suffer from more severe hematological symptoms^41^. Therefore, disturbance of the Ado pathway in DADA2 pathogenesis may directly or indirectly contribute to hematopoietic defects, highlighting the potential impact of Ado pathways disruption on the disease.

In vertebrates, eAdo signaling is mediated by four G-protein coupled cell-surface adenosine receptors (ARs): A_1_R, A_2a_R, A_2b_R and A_3_^42,43^. They are widely distributed and regulate multiple physiological processes either by activating (A_2a_R, A_2b_R) or inhibiting (A_1_R, A_3_R) the cAMP/PKA pathway^43^. ARs expression is tissue and cell-type specific making them potential targets for novel drugs^44–46^. Among ARs, A_2b_R is most likely to play a role in DADA2 context. Indeed, it directly interacts with ADA2, and a prolonged exposure to the ligand is required for its activation due to its low affinity for eAdo compared to the other AR activation^31,47^. Moreover, A_2b_R has been associated with several pathological mechanisms, such as inflammation, leukocyte and endothelial function, and specification and maintenance of hematopoietic stem progenitor cells (HSPCs)^48–54^. For instance, A_2b_R mediates the pro-inflammatory action of eAdo in monocytes and endothelial cells and reduces HSPC proliferation and mobilization^49–52,54,55^.

To gain insights into the mechanisms underlying DADA2 pathophysiology, we established a *cecr1b*-LoF zebrafish, which exhibits key characteristics of DADA2 patients, including neutropenia and inflammation. We analyzed in detail the hematopoietic processes in the *cecr1b*-deficient background and we found a defect in the emergence of definitive HSPCs from the endothelium. We identified a dysregulation of the A_2b_r receptor-mediated adenosine pathway in endothelial cells, which was the cause of the elevated inflammation and of the reduction of HSPC population. We also demonstrated the efficacy of in vivo administration of human ADA2 in correcting HSPCs and neutrophils populations, highlighting the translational potential of our model for studying DADA2.

## Results

### *cecr1b* deficiency recapitulates DADA2 human hematological and inflammatory phenotypes

**Fig. 1:**
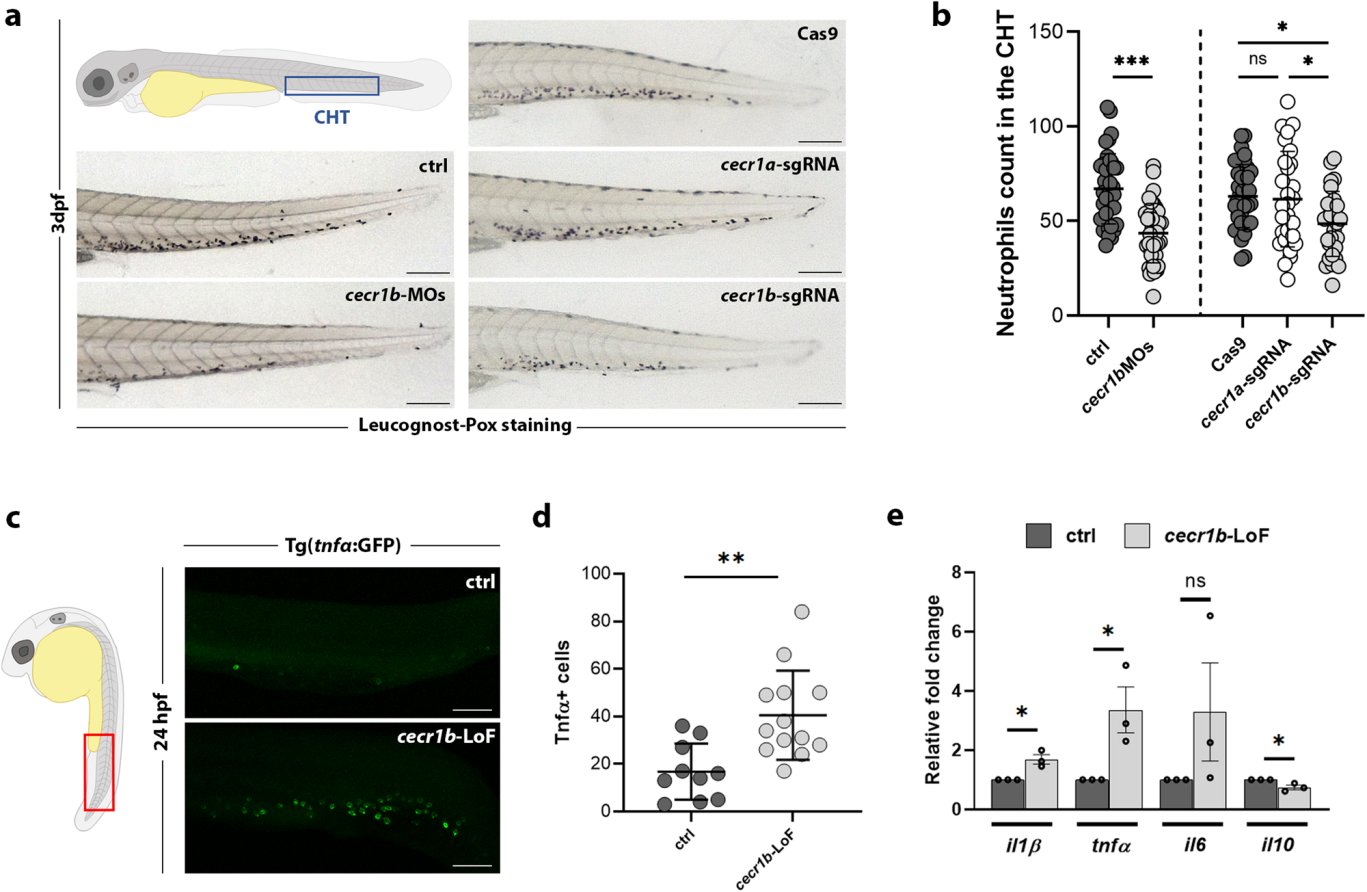
Generation and validation of the *cecr1b*-LoF zebrafish models. **a** Representative brightfield images of the CHT of 3 dpf embryos stained with the Leucognost-Pox colorimetric assay for neutrophils detection. Scale bar: 300 microns. **b** Quantification of neutrophils number in the CHT. Each dot represents the count of a single embryo (mean ± SD). Statistical significance was assessed by two-tailed, unpaired t-student test and Ordinary one-way ANOVA test with Tukey’s correction for *cecr1b*-MOs embryos (ctrl, n = 38 embryos; *cecr1b-*MOs n = 37 embryos) and *cecr1a/cecr1b*-sgRNA embryos (Cas9, n = 35 embryos; *cecr1a-*sgRNA, n = 28 embryos; *cecr1b*-sgRNA, n = 29 embryos), respectively; ***, *p* < 0.001; *, *p* < 0.05; ns, not significant (**c**) Representative confocal images of Tnfα expressing cells in the trunk-tail region of 24–26 hpf embryos. Magnification: 20X. Scale bar: 100 microns. **d** Count of Tnfα+ cells in the trunk region. Each dot in the graph represents the count of a single embryo (mean ± SD). Statistical significance was assessed by two-tailed, unpaired t-student test; **p < 0.002 (ctrl, n = 10 embryos; *cecr1b*-LoF, n = 13 embryos) (**e**) RT-qPCR results of pro-inflammatory (*il1β, tnfα, il6*) and anti-inflammatory (*il10*) cytokines in 3 dpf embryos (mean ± SEM). n = 3 biologically independent experiments. Statistical significance was assessed by a two-tailed unpaired t-student test; *p < 0.05, ns not significant.

Zebrafish (*Danio rerio*) possesses two *ADA2* orthologues: *cecr1a* (GRCz11, Chr25:16.689.633-16.700.924) and *cecr1b* (GRCz11, Chr4:5.225.552-5.240.128). *cecr1b* was identified as the paralogue with a conserved function to the human *ADA2* through morpholino-mediated knock-down and insertional mutagenesis experiments^1^. Knock-down of *cecr1b* led to neutropenia, mirroring the human phenotype, whereas *cecr1a* did not show any effect. We conducted additional knock-down and knock-out experiments to validate these results in our zebrafish model. For *cecr1b* knock-down, we injected a combination of *cecr1b*-ATG and *cecr1b*-splicing-blocking-morpholino^1^. Additionally, we employed the CRISPR-Cas9 technique using two sgRNAs targeting exon 5 (sgRNA1 and sgRNA2), which encodes a portion of the catalytic domain, to knock out *cecr1a* and *cecr1b* (Supplementary Fig. 1, Supplementary Fig. 2). Neutropenia served as the primary phenotype for comparing the *cecr1a* and *cecr1b* crispants (hereafter named *cecr1*-LoF). Myeloperoxidase positive (*mpx*+) mature neutrophils identified by Leucognost-Pox colorimetric assay were quantified in the caudal hematopoietic tissue (CHT) of 3 days post fertilization (dpf) embryos^56^. Neutrophil count was decreased in *cecr1b* morphants (*cecr1b*-MOs) and *cecr1b*-LoF, whereas no such reduction was observed in *cecr1a-*LoF, confirming the functional conservation between ADA2 and Cecr1b **(**Fig. 1a, b**)**. Therefore, all experiments were performed targeting the *cecr1b* paralogue only.

Given the high inflammation present in DADA2 patients, we analyzed this phenotype in the *cecr1b*-LoF zebrafish embryos using the transgenic line Tg(*tnfα*:GFP)^57^. We observed a significant increase of Tnfα+ cells in the trunk-tail region of *cecr1b*-LoF embryos from 24–26 hpf (Fig. 1c, d) and an increased expression of the pro-inflammatory cytokines *il1β*, *tnfα*, and *il6* by RT-qPCR (Fig. 1e). Conversely, the level of the anti-inflammatory cytokine *il10* was significantly reduced in *cecr1b*-LoF compared with ctrl zebrafish (Fig. 1e).

### *cecr1b* deficiency disrupts A_2_R signaling necessary for hemogenic endothelial cell specification and HSPC emergence

**Fig. 2:**
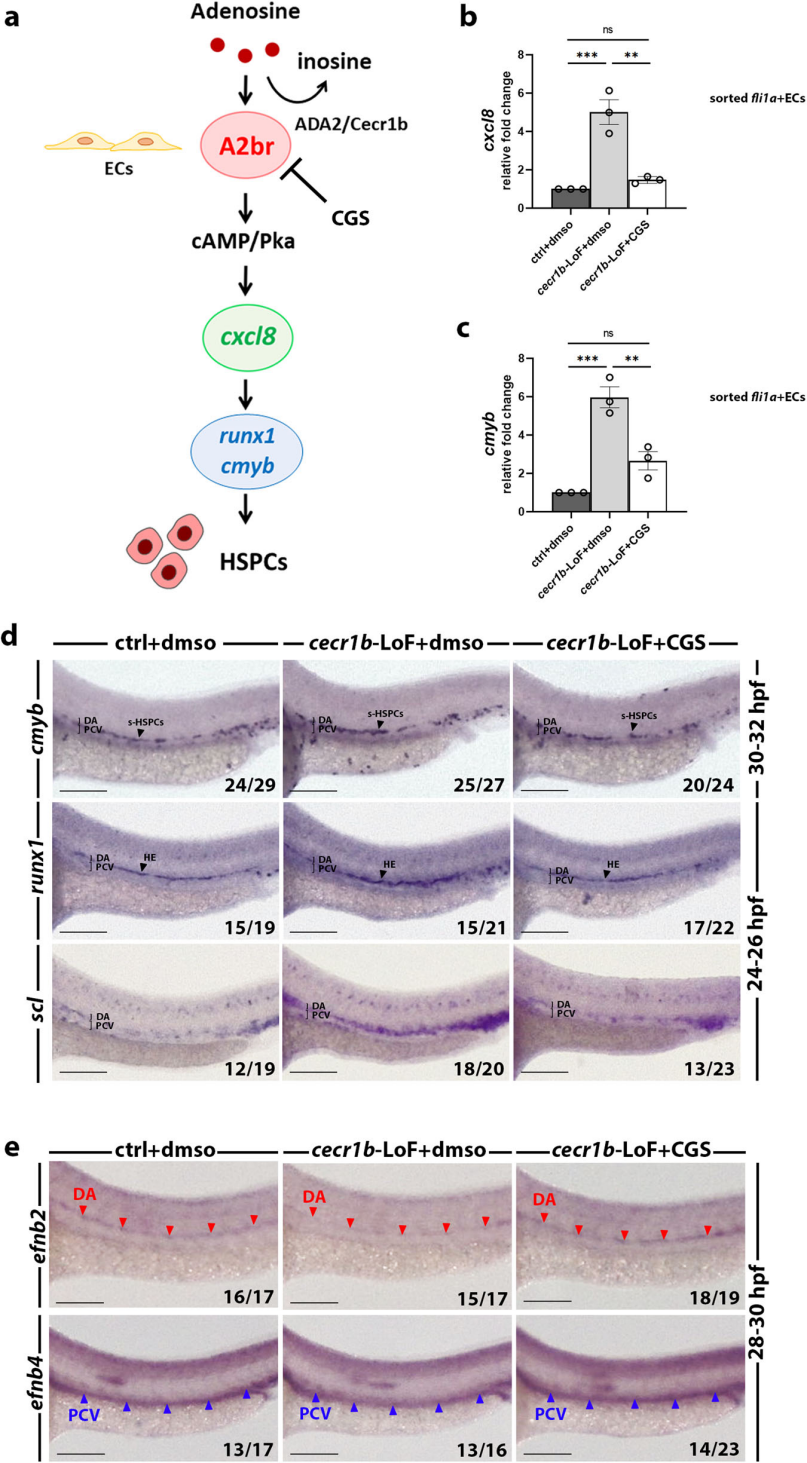
Analysis and modulation of the A_2b_R pathway, which is dysregulated in the endothelium of *cecr1b*-LoF embryos. **a** Schematic representation of the A_2b_R-dependent eAdo signaling pathway. A_2b_R activation in the endothelium leads to the expression of HSPC-specific genes (*runx1* and *cmyb*) through activation of the cAMP/Pka. Expression levels of (**b**) *cxcl8* and (**c**) *cmyb* measured by RT-qPCR in endothelial cells sorted (ECs) from 30–32 hpf Tg(*fli1a*:GFP)^*y1*^. Data are presented as mean ± SEM; n = 3 biologically independent experiments. Statistical significance was assessed by Ordinary one-way ANOVA test with Tukey’s correction; ***p < 0.001; **p < 0.002; ns, not significant. **d** Representative images of WISH staining for the HE markers *runx1* and *scl* in the trunk region of 24–26 hpf embryos and for the specifying HSPCs marker *cmyb* in 30-32 hpf embryos. DA dorsal aorta, PCV posterior cardinal vein, s-HSPCs specifying HSPCs, HE hemogenic endothelium. Numbers indicate the embryos belonging to the representative phenotype of each category, shown in the image. Scale bar = 100 microns. **e** WISH staining of the arterial (*efnb2*) and venous (*efnb4*) markers in the trunk region of 28–30 hpf embryos. DA dorsal aorta, PCV posterior cardinal vein. Red arrowheads indicate the region of the DA, blue arrowheads indicate the region of the PCV. Scale bar = 100 microns.

**Fig. 3:**
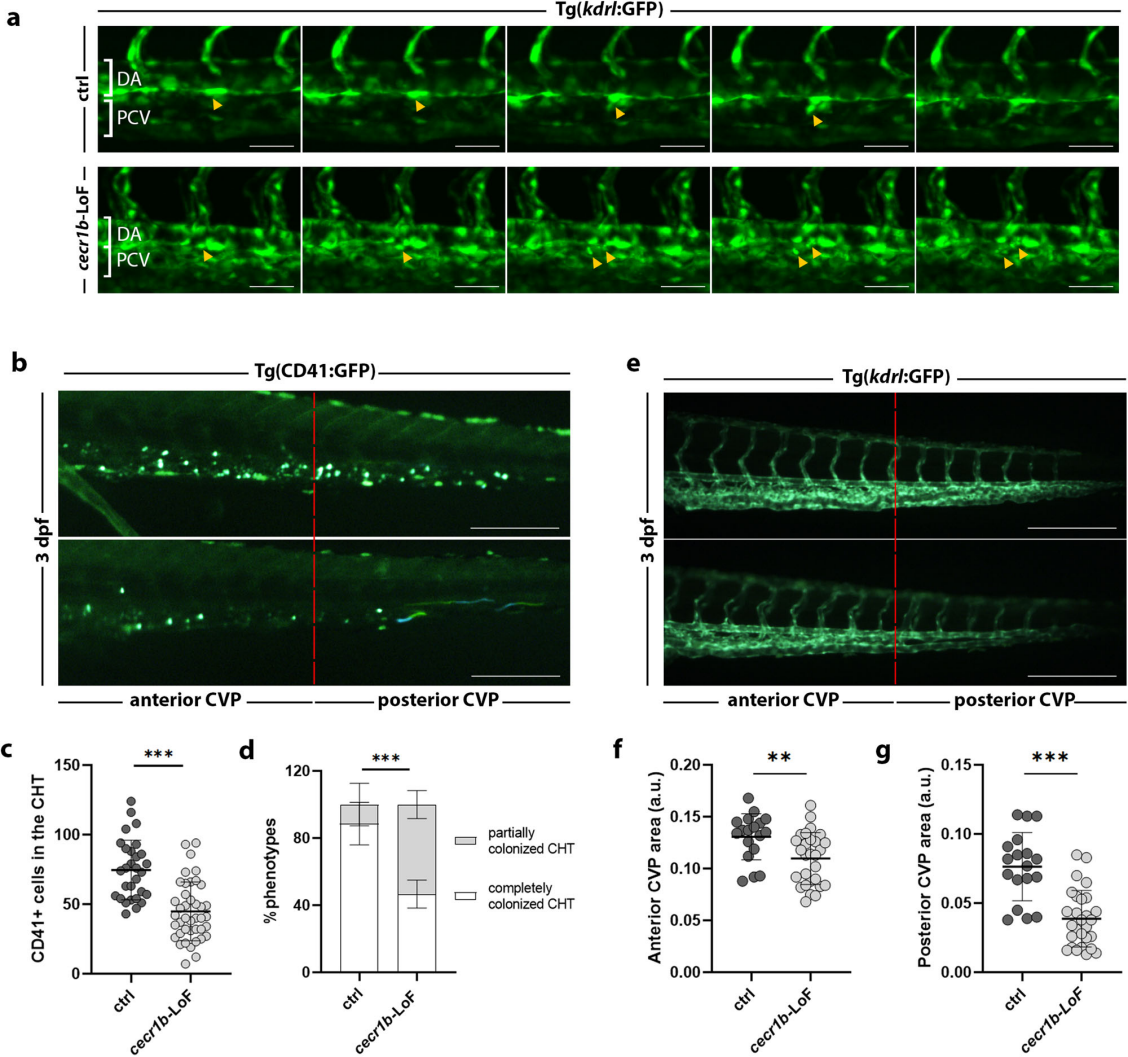
Defective HSPC emergence from HE and altered CHT colonization. **a** Sequence of images from time-lapse experiments on Tg(*kdrl*:GFP) ctrl and *cecr1b*-LoF embryos from 30 hpf. Yellow arrowheads indicate HSPCs emerging from the ventral wall of the dorsal aorta in the trunk region. DA dorsal aorta, PCV posterior cardinal vein. Magnification 20X, scale bar = 50 microns. **b** Representative fluorescence images of the CHT region of 3 dpf Tg(CD41:GFP) ctrl and *cecr1b*-LoF embryos. The red dotted line divides the CHT into the anterior and posterior parts. Scale bar = 150 microns. **c** Quantification graph of the CD41+ HSPC number (mean ± SD) in the CHT region of ctrl and *cecr1b*-LoF embryos at the stage of 3 dpf. Each dot represents the count of a single embryo. Statistical significance was assessed by a two-tailed, unpaired t-student test. ***p < 0.001. (ctrl, n = 29 embryos; *cecr1b*-LoF, n = 41 embryos). **d** Histograms showing the percentage of embryos with completely colonized (white) or partially colonized (gray) CHT. Data are presented as the mean ± SD of independent experiments. n = 3 biologically independent experiments. Statistical significance was assessed with the Chi-square test (Fisher’s exact test, confidence interval 95%). ***p < 0.001. **e** Representative fluorescence images of the CVP region of 3 dpf Tg(*kdrl*:GFP). The red dotted line divides the CVP region into the anterior and posterior parts. Quantification graph of the GFP+ area in the (**f**) anterior and (**g**) posterior part of the CVP. Each dot represents the count of a single embryo. Data are presented as mean ± SD. Statistical significance was assessed with an unpaired t-student-test. ***p < 0.001; **p < 0.002 (ctrl, n = 18 embryos; *cecr1b*-LoF, n = 28 embryos).

To gain a better understanding of the origin of *cecr1b*-dependent neutropenia, we focused on the HSPC population, which represents the earliest precursor of neutrophils. In vertebrates, HSPCs emerge from the hemogenic endothelial cells through endothelial to hematopoietic transition (EHT)^19,58,59^. The EHT process is regulated by a complex network of molecular interactors, which include eAdo signaling mediated by A_2b_R. A_2b_R is the sole adenosine receptor expressed in the hemogenic endothelium (HE) of the developing zebrafish embryo^60–62^. It is known that the activation of the A_2b_R/cAMP/PKA/*cxcl8* pathway within the HE triggers initiation of the hematopoietic transcriptional program, leading to the expression of specific markers for HSPCs specification, such as *runx1* and *cmyb* (Fig. 2a)^60^. Currently, the mechanisms responsible for regulating eAdo levels and mediating the physiological activation of the A_2b_R pathway remain unidentified. Considering the ability of ADA2 to degrade eAdo, we investigated whether *cecr1b* deficiency could interfere with the A_2b_R pathway, leading to alterations in the HE and HSPC specification^25,31^. We sorted GFP^+^ endothelial cells from Tg(*fli1a*:GFP)^*y1*^ ^63^ embryos at 32-34 hpf to evaluate the expression of the two genes downstream of A_2b_R signaling: *cxcl8* and *cmyb*^60^ (Supplementary Fig. 3). Both genes were overexpressed in *cecr1b*-LoF embryos, indicating a hyperactivation of the A_2b_r pathway during EHT^60^ (Fig. 2b, c). Treatment of the *cecr1b*-LoF embryos with the A_2_R antagonist CGS-15943 (referred to as CGS) successfully reduced the expression of *cxcl8* and *cmyb* genes, confirming the dependence of their upregulation on eAdo signaling (Fig. 3b, c, Supplementary Fig. 4). Dysregulation of this pathway was further confirmed by expression analyses on whole embryos (Supplementary Fig. 4). Notably, treatment of the *cecr1b*-LoF embryos with H89, a selective PKA inhibitor, also reduced the expression of A_2b_R-related genes. In contrast, the administration of the eAdo analog NECA further enhanced it, confirming the involvement of the pathway in this phenotype (Supplementary Fig. 4). We evaluated the expression of *scl* and *runx1* genes, both essential for HE specification via whole mount in-situ hybridization (WISH) analyses^64–66^. Consistent with the A_2b_R endothelial hyperactivation, we observed an increase in both *scl* and *runx1* signals in the ventral wall of the dorsal aorta - where the HE is located – of 24-26 hpf *cecr1b*-LoF embryos (Fig. 2d). Similarly, the *cmyb* signal in the HE was increased in *cecr1b*-LoF embryos compared to the controls. Treatment with CGS rescued the upregulation of all these markers (Fig. 2d, Supplementary Fig. 4). Since *runx1* expression in HE down-regulates the arterial program enabling HSPC differentiation, we decided to analyse arterial-venous specification in *cecr1b*-LoF^67^. The arterial marker *efnb2* was reduced in *cecr1b*-LoF, as expected by *runx1* increased expression. Similarly, the venous marker *efnb4* was slightly diminished, suggesting improper specification of the axial vessels (Fig. 2e). The modulation of the A_2_R pathway through CGS administration also restored these defects, further demonstrating the role of the A_2b_R pathway in the endothelium (Fig. 2e)^68^.

The formation of primitive HSCs is strictly dependent on primitive hematopoiesis. Therefore, we analyzed a possible contribution of primitive myeloid cells to the increased expression of *runx1* and *cymb*. We evaluated the expression of *pu.1*, marker of myeloid precursors, during somitogenesis and the expression of *l-plastin*, mature primitive myeloid cells, at 24 hpf. The expression of both markers were reduced in the *cecr1b*-LoF embryos in comparison to controls and was rescued through CGS administration (Supplementary Fig. 5), thus the reduction of primitive myeloid cells was in contrast to the increased expression of *runx1* and *cmyb*. This result suggests that the endothelial hyperactivation/inflammatory state is a principle driver of the phenotype.

To find out whether the increase in the *runx1* and *cmyb* markers in the HE was linked to an impairment in HSPC number, we took advantage of the double transgenic line TgBAC(*runx1*:Citrine);Tg(*kdrl*:mCherry)^69^. We performed in vivo time point analyses between 26 and 34 hpf to directly look at specifying HSPCs in the *cecr1b*-deficient background. Despite the overall increase in *runx1* expression, the total number of ventral-dorsal-aorta-associated Runx1-citrine+ HE cells was reduced in *cecr1b*-LoF embryos starting from 30 hpf. CGS administration corrected Runx1-citrine+ specifying HSPC number (Supplementary Fig. 6).

To better characterize HSPC emergence and budding from the HE, we performed in-vivo time-lapse analyses in Tg(*kdrl:GFP*)^70^ embryos from 30 to 36 hpf. (Fig. 3a and Supplementary Movie 1, 2). Normally, from 32 hpf onwards the endothelial cell contracts, initiates EHT, buds from the aorta floor and enters the blood flow to seed the CHT^19^. In *cecrb*-LoF, we observed abortive EHT events with HSPCs breaking into pieces as they bud from the HE (Fig. 3a and Supplementary Movie 1, 2).

Accordingly, using the transgenic line Tg(CD41:GFP) labeling HSPCs^71^, we noted a significant reduction of CD41+cells seeding in the CHT of 3 dpf *cecr1b*-LoF larvae compared to controls (Fig. 3b, c). To exclude possible defects in HSPC migration, from the HE to the CHT, we performed time-point WISH analyses for the *cmyb* marker from 36 hpf to 3 dpf. Staining for *cmyb* in *cecr1b*-LoF embryos revealed a migration kinetics to the CHT similar to that of controls (Supplementary Fig. 7). However, *cecr1b*-LoF embryos exhibited a partial and atypical CHT colonization, with most of the HSPCs accumulating in the anterior region (Fig. 3d). Proper homing and expansion of HSPCs in the CHT is ensured by the caudal vein plexus (CVP), which provides a supportive and protective environment for HSPCs^72–74^. Analyses with the endothelial-specific Tg(kdrl:GFP)^70^ transgenic *cecr1b*-LoF embryos revealed that, despite the presence of circulation, they showed an altered architecture of the CVP, which explained the abnormal CHT colonization (Fig. 3e–g). The CVP vascular net appeared reduced both in the anterior region and, to a greater extent, in the posterior one of *cecr1b*-LoF embryos, with some areas totally lacking vascularization (Fig. 3e–g).

**Fig. 4:**
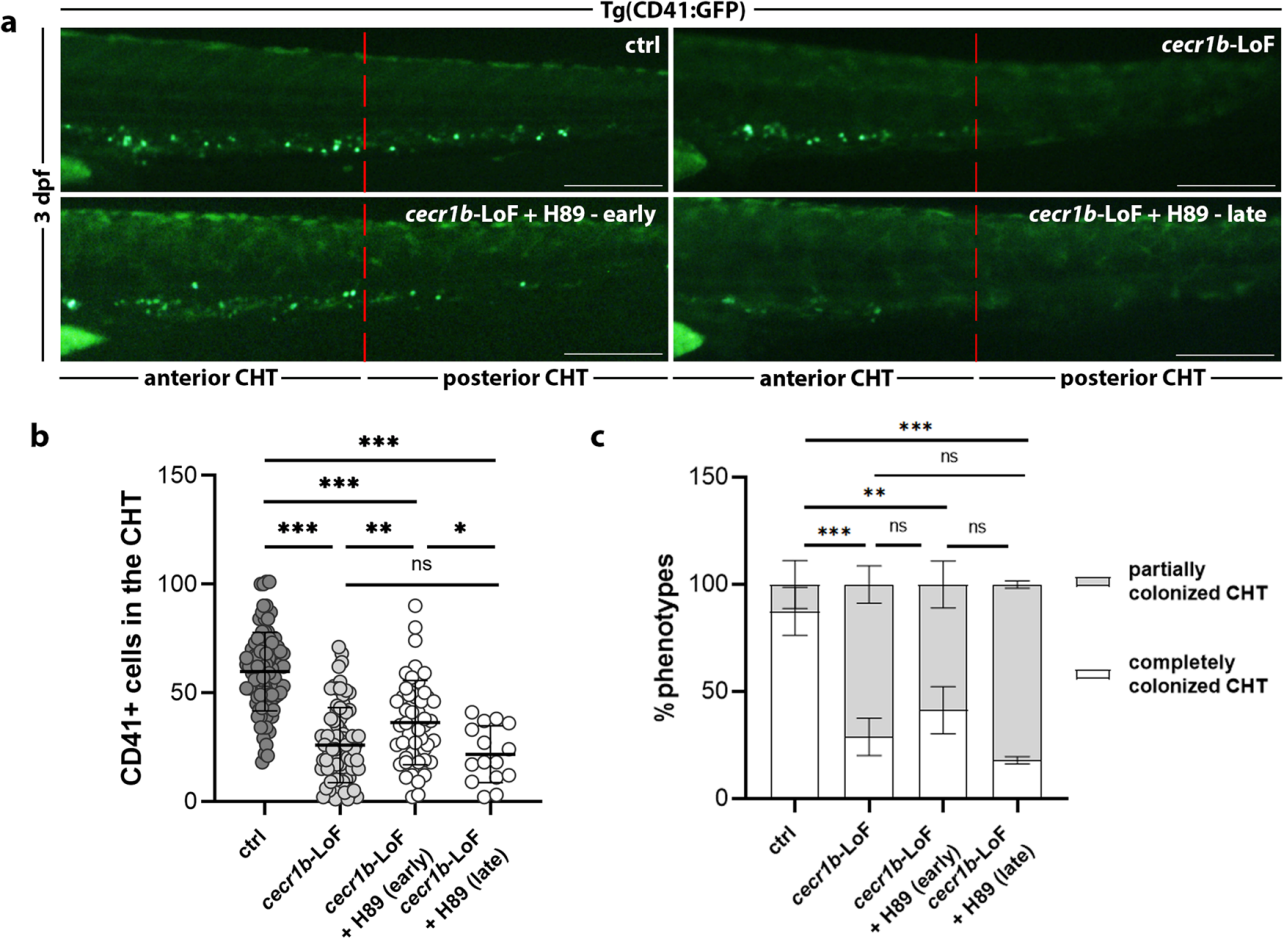
Analysis of the defective HSPC population in the CHT of *cecr1b*-LoF embryos and its correction through A_2b_R modulation. **a** Representative fluorescence images of the CHT region of 3 dpf Tg(CD41:GFP) embryos. Scale bar: 150 microns. **b** Quantification of CD41+ HSPCs in the CHT in *cecr1b*-LoF embryos treated with H89 from early or late time. Each dot represents the count of a single embryo. Data are presented as mean ± SD. Statistical significance was assessed by Ordinary one-way ANOVA test with Tukey’s correction; ***, *p* < 0.001; **, *p* < 0.002; *, *p* < 0.05; ns not significant (ctrl, n = 96 embryos; *cecr1b*-LoF, n = 82 embryos; *cecr1b*-LoF+H89 (early), n = 52 embryos; *cecr1b*-LoF+H89 (late), n = 15 embryos). **c** Histogram showing the proportion of embryos showing complete or defective CHT colonization in the different experimental conditions (mean of biological experimental replicates±SD). n = 3 biologically independent experiments. Statistical significance was assessed by multiple Chi-Square analyses (Fisher’s exact test, confidence interval 99%); ***p < 0.001; **p < 0.002; *p < 0.05; ns not significant.

We examined whether modulation of the A_2b_R pathway could effectively correct defects in HSPCs population. H89 treatment effectively restored CD41+ HSPC number in *cecr1b*-LoF embryos (Fig. 4a–c). Interestingly, the rescue was efficient only when H89 treatment was administered from 24 hpf (early), prior to HSPC emergence from the HE, but not from 36 hpf (late) (Fig. 4a–c). These findings emphasize that early modulation of eAdo signaling can rescue *cecr1b*-dependent hematopoietic defects, highlighting the critical role of the A_2b_R pathway during the specific time window when HSPC emerge from the HE.

### HSPC defects are recovered by reducing inflammation in *cecr1b*-LoF embryos

**Fig. 5:**
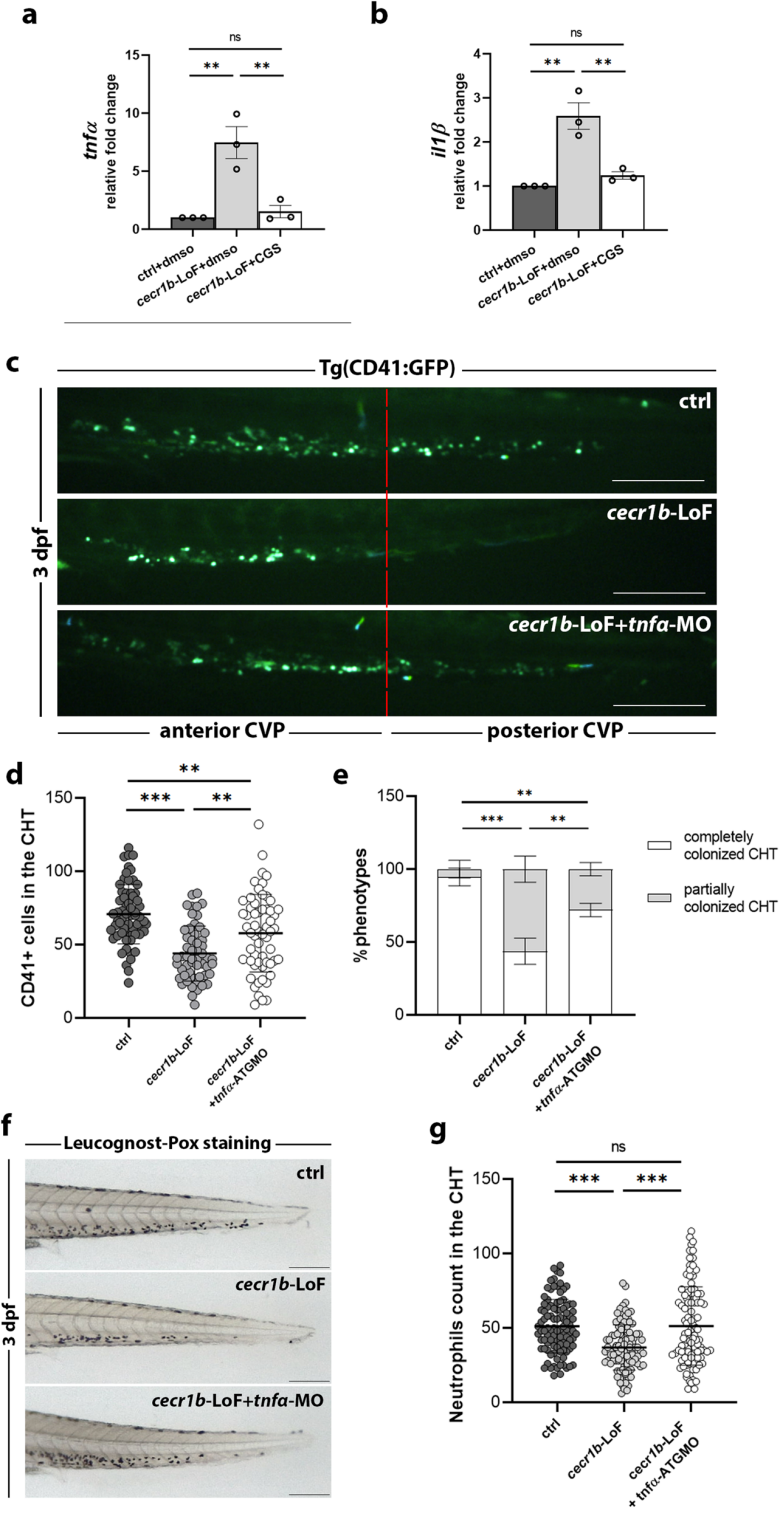
Analysis of the defective HSPC population in the CHT of *cecr1b*-LoF embryos and its correction trough *tnfα* modulation. RT-qPCR expression levels of (**a**) *tnfα* and (**b**) *il1β* measured in endothelial cells sorted from 30-32 hpf Tg(*fli1a*:GFP) ctrl and *cecr1b*-LoF embryos untreated or treated with CGS. Data are presented as mean ± SEM. n = 3 biologically independent experiments. Statistical significance was assessed by Ordinary one-way ANOVA test with Tukey’s correction; **p < 0.002; ns, not significant. **c** Representative fluorescence images of the CHT region of 3 dpf Tg(CD41:GFP) embryos belonging to the different experimental categories. Scale bar: 150 microns. The red dotted line divides the CHT region into the anterior and posterior parts. **d** Quantification graph of CD41+ HSPCs in the CHT of ctrl 3 dpf embryos belonging to the different experimental categories. Each dot represents the count of a single embryo. Data are presented as mean ± SD. Statistical significance was assessed by Ordinary one-way ANOVA test with Tukey’s correction; ***, *p* < 0.001; **, *p* < 0.002; (ctrl, n = 62 embryos; *cecr1b*-LoF, n = 48 embryos; *cecr1b*-LoF+tnfα-ATGMO, n = 58 embryos); **e** Histogram showing the proportion of embryos with complete or defective CHT colonization in the different experimental conditions (mean ± SD). n = 3 biologically independent experiments. Statistical significance was assessed by multiple two-sided Chi-Square analysis (confidence interval 99%); ***p < 0.001; *p < 0.05. Representative bright-field images (**f**) and quantification graph (**g**) of neutrophils stained with the Leucognost-Pox colorimetric assay in the CHT of 3 dpf embryos. Scale bar: 300 microns. Each dot represents the count of a single embryo. Data are presented as mean ± SD. Statistical significance statistical significance was assessed by Ordinary one-way ANOVA test with Tukey’s correction; ***p < 0.001, ns, not significant (ctrl, n = 100 embryos; *cecr1b*-LoF, n = 107 embryos; *cecr1b*-LoF+tnfα-ATGMO, n = 104 embryos).

HSPC emergence is strictly dependent on inflammatory signaling in the HE^75^ and, notably, recurrent phenotypes of DADA2 patients are increased inflammation and vasculitis. To investigate whether A_2b_R pathway hyper-activation induces endothelial inflammation, we measured the expression of pro-inflammatory cytokines in endothelial cells sorted from Tg(*fli1a*:GFP)^*y1*^ ^63^ *cecr1b*-LoF embryos. We found that *il1β* and *tnfα* expression increased in *cecr1b*-LoF endothelial cells, was successfully corrected by modulation of the A_2b_R pathway (Fig. 5a, b). Similarly, CGS administration mitigated the systemic inflammatory state of *cecr1b*-LoF embryos (Supplementary Fig. 8). To investigate the potential link between inflammation and the reduction of HSPC population, we injected a *tnfα*-ATG morpholino (*tnfα*-ATG-MO) to modulate the inflammatory state of *cecr1b*-LoF embryos. Downregulation of *tnfα* recovered HSPC number and improved CHT colonization (Fig. 5c–e). Similarly, *tnfα* modulation restored neutrophil population of *cecr1b*-LoF embryos (Fig. 5f, g). Overall, these data indicate that endothelial inflammation is directly involved in the generation of hematopoietic defects of *cecr1b*-LoF embryos.

### Defective hematopoiesis can be rescued in *cecr1b*-LoF embryos by supplementation with recombinant human ADA2

**Fig. 6:**
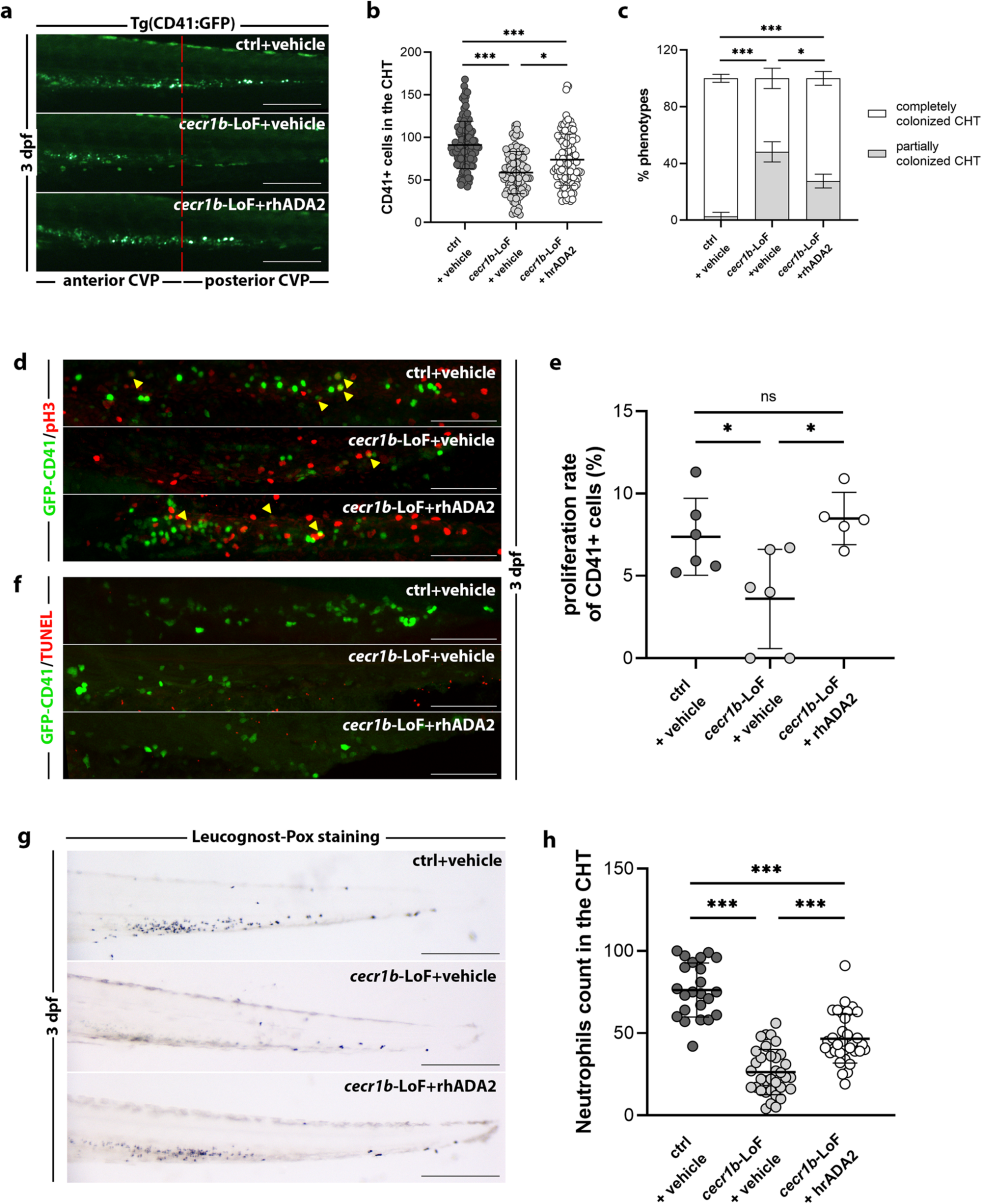
Recovery of the *cecr1b*-dependent hematopoietic phenotypes through supplementation of the rhADA2. **a** Representative fluorescence images of the CHT region of 3 dpf Tg(CD41:GFP) embryos belonging to the different experimental conditions. The red dotted line divides the CHT region into the anterior and posterior parts. Scale bar: 150 microns. **b** Quantification graphs of CD41+HSPC number in the CHT region of the embryos. Each dot represents the count of a single embryo (mean ± SD). Statistical significance was assessed by Ordinary one-way ANOVA test with Tukey’s correction; ***p < 0.001; *p < 0.05 (ctrl, n = 109 embryos; *cecr1b*-LoF, n = 109 embryos; *cecr1b*-LoF+rhADA2, n = 107 embryos). **c** Histogram showing the proportions of embryos showing complete or defective CHT colonization (mean ± SD). n = 5 biologically independent experiments. Statistical significance was assessed by multiple two-sided Chi-Square analysis (Fisher’s exact test, confidence interval 99%); ***p < 0.001; *p < 0.05. **d** Representative confocal images of the CHT region of 3 dpf embryos stained for CD41/GFP and the proliferation marker pH3 with double immuno-fluorescence. Yellow arrowheads indicate double CD41-GFP+/pH3+ cells. Magnification: 20X. Scale bar: 100 microns. **e** The proliferation rate was estimated as the number of double CD41-GFP+/pH3+ cells normalized on the total number of CD41-GFP+ in the CHT of each embryo. Statistical significance was assessed by Ordinary one-way ANOVA test with Tukey’s correction; *p < 0.05, ns not significant (ctrl, n = 6 embryos; *cecr1b*-LoF, n = 6 embryos; *cecr1b*-LoF+rhADA2, n = 5 embryos). **f** Representative confocal images of the CHT region of 2*.*5 dpf embryos stained with the apoptotic TUNEL assay. Magnification: 20×. Scale bar: 100 microns (ctrl, n = 4 embryos; *cecr1b*-LoF, n = 4 embryos; *cecr1b*-LoF, n = 4 embryos). **g**, **h** Representative images and quantification of neutrophils stained with the Leucognost-Pox colorimetric assay in the CHT of 3 dpf embryos. Scale bar: 300 microns. Each dot in the graph represents the count of a single embryo (mean ± SD). Statistical significance was assessed by Ordinary one-way ANOVA test with Tukey’s correction; ***p < 0.001 (ctrl, n = 24 embryos; *cecr1b*-LoF, n = 34 embryos; *cecr1b*-LoF+rhADA2, n = 36 embryos).

We sought to determine whether ADA2 supplementation could correct the abnormal hematological characteristics observed in *cecr1b*-LoF embryos. The recombinant human ADA2 (rhADA2) protein was administered via systemic intra-vascular injection to *cecr1b*-LoF Tg(CD41:GFP)^71^ embryos at 26-28 hpf (Fig. 6a–c). A dose-dependent improvement in the HSPC number and CHT colonization was observed at 3 dpf, demonstrating that ADA2 supplementation can correct the defective hematological phenotypes observed in *cecr1b*-LoF embryos (Supplementary Fig. 9). Furthermore, we explored the possibility that ADA2 promotes the proliferation and survival of HSPCs, given its proposed function as a growth factor^26^. The proliferation rate of HSPCs was assessed by quantifying the number of double-positive GFP+/p-H3+ cells among the total GFP+ cells in the CHT of *Tg*(CD41:GFP)^71^ embryos at 3 dpf. In most *cecr1b*-LoF embryos, HSPCs were nearly absent. In contrast, following treatment with rhADA2, HSPC number was restored (Fig. 6d, e). Moreover, the TUNEL assay showed no differences between HSPC number in the CHT of *cecr1b*-LoF and control Tg(CD41:GFP) embryos, confirming that their reduction was due to early cell death during EHT but not during CHT colonization (Fig. 6f). The rhADA2-mediated restoration of the HSPC compartment was accompanied by a partial correction of neutropenia as measured by *Mpx M Mpx*+ neutrophil staining (Fig. 6g, h).

These findings showed that rhADA2 promotes HSPC emergence and differentiation, ultimately ameliorating the neutropenic phenotypes in *cecr1b*-LoF embryos.

## Discussion

This study reveals that *cecr1b* deficiency in zebrafish accurately mirrors human DADA2 traits, including neutropenia, vascular dysfunction, and inflammation, underscoring the functional similarity between ADA2 and Cecr1b. DADA2 patients show a strong genotype-phenotype correlation. Vasculitis and systemic inflammation are usually associated with missense mutations retaining residual enzymatic function, while hematological impairments are typically linked to mutations with minimal catalytic activity or complete loss-of-function^41^. The broad phenotypic spectrum in our zebrafish model may stem from our knock-out (KO) strategy, which generates mosaicism and various mutations within the same organism due to the error-prone DNA repair mechanisms following Cas9-mediated DNA nicks in different cells. Such a state of genetic mosaicism brings the advantage of approaching what is reported in DADA2 patients, who rarely have null alleles in homozygosity but, in most cases, are compound heterozygotes (^5,76,77^). Moreover, there are documented cases of patients with heterozygous missense mutations and carriers who exhibit mild symptoms, suggesting that the disease severity may be dose-dependent. Our strategy targeting the *cecr1b* exon 5 for deletion was designed to disrupt the catalytic domain and cause mutations that could affect ADA2 function at varying degrees. On the other hand, our knock out approach could lead to a general decrease in ADA2 activity, affecting several domains rather than targeting one specific region.

We found that *cecr1b* deficiency disrupts A_2b_R-mediated adenosine signaling, causing increased inflammation with consequent vascular dysfunction and improper specification and function of the hemogenic endothelium, ultimately leading to defective HSPC emergence, expansion, and differentiation. Maintaining balanced eAdo levels is essential for proper hematopoiesis. Zebrafish studies by ref. ^60^ demonstrated that activation of the A_2b_R/cAMP/PKA/*cxcl8* pathway in the hemogenic endothelium triggers the expression of *runx1* and *cmyb*, crucial for HSPC emergence during embryo development^60^. Our research revealed the role of Cecr1b as a key enzyme regulating eAdo levels and ensuring appropriate A_2b_R signaling. When Cecr1b was functionally absent, the A_2b_R pathway was hyperactivated, inducing inflammatory, endothelial, and hematopoietic defects. Remarkably, chemical inhibition of the A_2_R/PKA axis rescued all the phenotypes, emphasizing the crucial role of this pathway during early developmental stages in zebrafish. DADA2 patients are born without apparent disease signs, likely due to ADA2 transfer from carrier mothers through the placenta, controlling inflammation and supporting normal hematopoietic development. The disease manifests during early childhood, especially in patients with severe mutations resulting in complete enzyme activity loss^41^. The progression of symptoms tends to worsen with age, indicating a complex interplay of factors beyond mutation severity. Notably, we observed diverse disease evolution in homozygous twins, with one experiencing rapid deterioration, requiring allogeneic bone marrow transplantation, while the other maintaining stable neutrophil levels under low-dose G-CSF treatment^16^.

**Fig. 7:**
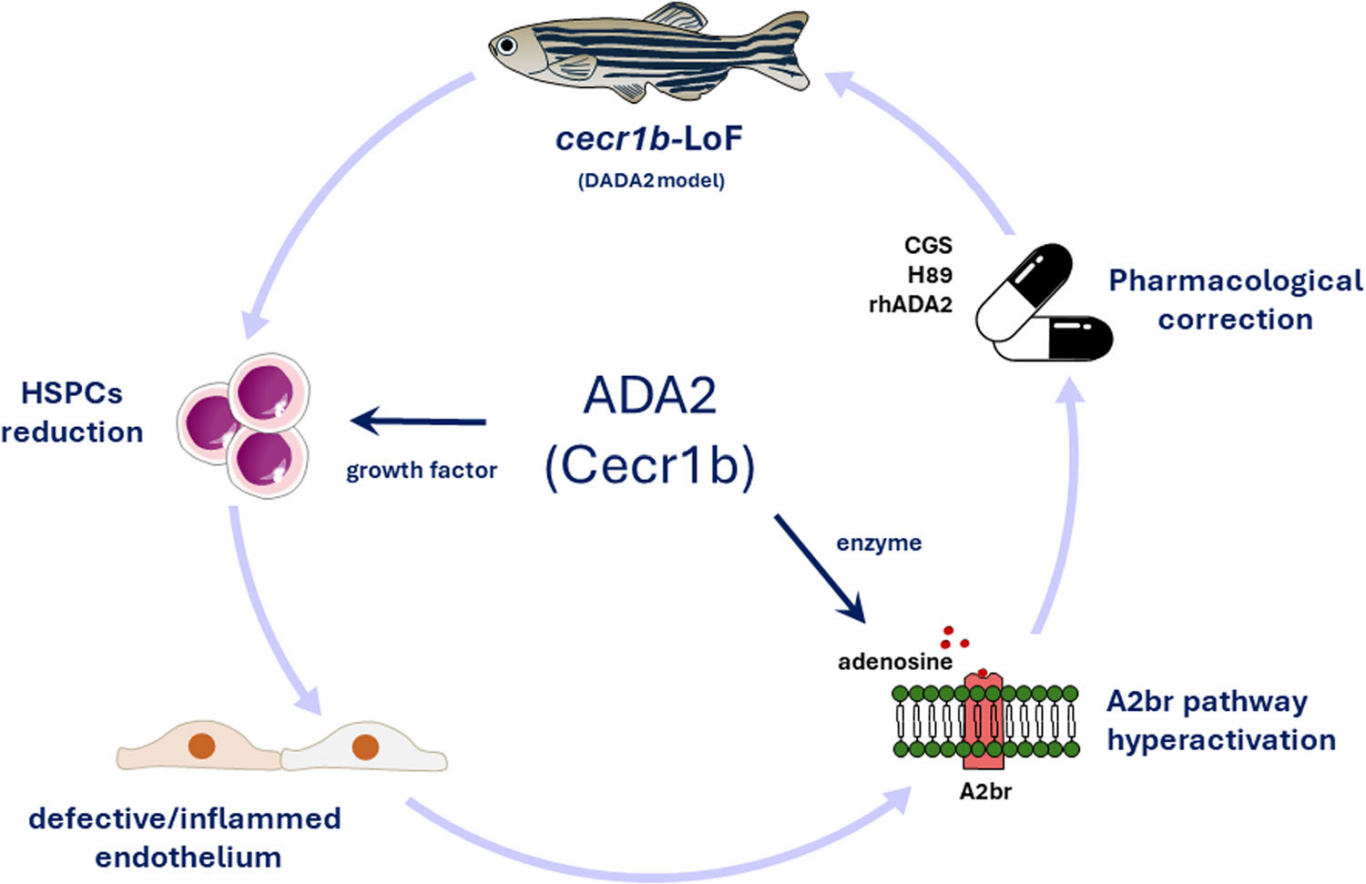
ADA2 has a role in regulating inflammation and HSPC emergence via the A_2b_R pathway in the *cecr1b*-LoF zebrafish model. Schematic representation of the function of ADA2 validated in the *cecr1b*-LoF zebrafish model.

Our DADA2 zebrafish model allows the investigation of pathogenetic mechanisms and relevant pathways that cannot be easily studied in patients or rodents since the latter lacks the *ADA2* gene (Fig. 7). Our finding reveal that Cecr1b serves as a key regulator of the eAdo-A_2b_r pathway, impacting hematopoiesis in zebrafish embryos. This revelation not only deepens our comprehension of the critical molecular pathways governing hematopoiesis but also highlights potential therapeutic strategies for addressing ADA2 deficiency in humans. Carmona-Rivera et al.’s initial finding, which reported elevated adenosine levels in the plasma of DADA2 patients, though requiring further validation in a broader patient cohort^40^, underscores the necessity to investigate whether disruptions in adenosine pathways underlie the hematopoietic symptoms observed in DADA2 cases. Should this be the case, developing targeted therapies that specifically address the disrupted eAdo-A_2b_r pathway in DADA2 patients becomes crucial. Increased eAdo levels in ADA2 deficiency, potentially exacerbating hematopoietic issues via the A_2b_/PKA pathway, suggests that therapeutic approaches designed to either adjust eAdo concentrations or inhibit its interaction with the A_2b_ receptor could prove beneficial. Therefore, options such as A_2b_ receptor blockers or PKA pathway inhibitors may serve as viable strategies to alleviate the hematopoietic abnormalities linked to ADA2 deficiency.

In recent years, the role of inflammatory signals within the bone marrow microenvironment has gained attention as critical factors influencing the generation and differentiation of HSPCs during steady-state hematopoiesis and in response to stress or injury^78^. Our study revealed the role of Cecr1b in regulating the inflammatory state. Indeed, *cecr1b*-deficient embryos displayed systemic inflammation and vasculitis as DADA2 patients. To delve deeper into the molecular mechanisms involved in the generation of such phenotypes, we explored the interplay between the A_2b_R/PKA pathway and inflammation, and we successfully rescued the inflammatory phenotype observed in *cecr1b*-deficient zebrafish embryos by inhibiting it. A_2b_R-mediated purinergic signaling also inhibits the mobilization, homing, and engraftment of cultured HSPCs^54,79,80^. This process involves the inflammasome, a multiprotein complex responsible for activating pro-inflammatory cytokines, such as IL-1β and IL-18, thus leading to an inflammatory response that negatively affects HSPC migration and integration into the target tissue^54,79,80^. Since TNFα and IL-1β have a pivotal role in the emergence of HSPCs by inducing the expression of RUNX1 in the hemogenic endothelium, their production should be tightly regulated. Indeed, prenatal exposure to pro-inflammatory cytokines causes persistent impairment of the immune compartment as chronic inflammation is deleterious for maintenance of adult HSPCs^81–83^. Consistent with the heightened expression of *il1β* and *tnfα* observed in *cecr1b*-LoF embryos, our data reveal an initial increase in *scl*, *runx1*, and *cmyb* expression, associated with a defective arterial-venous specification of the axial vessels. Cecr1b deficiency then seems to force the hemogenic endothelium toward the hematopoietic fate, resulting in overexpression of these hematopoietic markers. Despite the increase in hemogenic cell markers, HSPCs fail to undergo the EHT properly and die just after budding from the endothelium. The additional defects in the vascular architecture in the caudal hematopoietic tissue, where HSPCs expand and differentiate, further affect this cell population. Moreover, in cell-culture experiments, the overexpression of specific *RUNX1* isoforms before the specification of hemogenic endothelial cells prevents the transition of the mesoderm to HE, with consequent loss of HSPCs^84–86^.

Besides the characterization of the DADA2-like phenotypes in *cecr1b*-LoF zebrafish, we also tested the potential of an enzyme-replacement therapy in recovering the hematopoietic defects. This therapy is currently not used to treat patients due to the very short half-life of the protein^5^. In zebrafish, the vascular, immune, and hematopoietic developmental processes occur rapidly, and the respective systems and organs are completely functional within a few days after fertilization. In this context, the supplementation of the human recombinant protein has proved successful in correcting HSPC defects. This finding suggests the potential for using enzyme replacement therapy in patients. We also investigated the therapeutic potential of neutralizing TNFα activity to alleviate excessive inflammation in *cecr1b*-LoF zebrafish employing a TNFα morpholino. TNFα morpholino treatment effectively restored normal neutrophil levels in the *cecr1b*-LoF zebrafish. It is worth noting that anti-TNF therapy, used in patients to reduce inflammation and prevent strokes, does not ameliorate neutropenia^5,9^. This discrepancy could be attributed to patients receiving treatment when they are already neutropenic, and considerable hematopoietic damage may have already occurred. Nonetheless, these promising outcomes from both ADA2 enzyme replacement therapy and the exploration of anti-TNFα interventions lay the groundwork for further research, bringing hope for improved treatments that enhance the quality of life for patients with DADA2 and related disorders.

In summary, our study conducted with the DADA2 zebrafish model sheds light on the intricate relationship between *cecr1b*, inflammatory signals, and the emergence of HSPCs. We uncovered the role of Cecr1b in regulating eAdo levels and ensuring proper A_2b_R signaling. The promising results obtained from ADA2 enzyme replacement therapy and the potential of anti-TNFα interventions offer encouraging prospects for developing more effective therapeutic strategies and ultimately improving the quality of life for patients affected by DADA2.

## Methods

### Animals’ husbandry

Adult zebrafish were bred in accordance with internationally (EU Directive 2010/63/EU; European recommendations) and national recognized guidelines (Italian decree 4th March 2014, n. 26), which focus on the protection of animals used for scientific research. All experiments were performed on zebrafish embryos and larvae within 5 days post fertilization. Within this time window zebrafish in not considered as an animal for experimentation according to the Italian D.Lsg 26/14. All the experiments were conducted minimizing stress and pain with the use of proper analgesic/anesthetic when needed. The zebrafish were raised in tanks equipped with a recirculating water system, maintaining a constant temperature of 28 °C. Additionally, the zebrafish were subjected to a 14-h light and 10-h dark cycle to establish a regular light/dark cycle.

To obtain fertilized eggs, breeding pairs were placed in dedicated breeding tanks, and the eggs were collected following natural spawning. The embryos were then transferred to standard 90 mm × 15 mm Petri dishes containing fresh E3 medium (50× stock solution: 73.0 g of NaCl, 3.15 g of KCl, 9.15 g of CaCl2, and 9.95 g of MgSO4 in 5 L of distilled H2O) and maintained at a temperature of 28 °C for up to 24 hpf. E3 stock solution was diluted in distilled water supplemented with 200 μl of 0.05% methylene blue. From 24 hpf onwards, the embryos were grown in an E3 medium supplemented with 0.003% 1‐phenyl‐2‐thiourea (PTU, Sigma‐Aldrich, Saint Louis, MO) to prevent pigmentation. Before conducting analyses, the chorions were mechanically removed using thin needles or tweezers and then anesthetized with a 0.016% tricaine solution (ethyl 3‐aminobenzoate methanesulfonate salt; Sigma‐Aldrich). The following zebrafish lines were used for the experiments: AB wild-type (European Zebrafish Resource Center, EZRC), Tg(CD41:GFP)^71^, Tg(*fli1a*:GFP)^*y1*^^63^, Tg(*tnfa*:GFP)^57^, Tg(*kdrl*:GFP)^70^, and Tg(*runx1*:citrine);Tg(*kdrl*:mCherry)^69^.

### Knock-down and knock-out techniques

The knock-down of *cecr1b* was achieved using morpholino oligonucleotides obtained from Gene Tools (Philomath, OR). A mixture of two *cecr1b* morpholinos^1^ was injected into 1-2 cell embryos at a dose of 0.15 pmol per embryo for each morpholino (refer to Supplementary Table 1 for sequences). The knock-down efficacy of the *cecr1b* splicing morpholino (*cecr1b*-sMO) was validated by RT-PCR. Total RNA was extracted from 24 hpf control and *cecr1b*-sMO injected embryos, followed by retro-transcription and pre-amplification using the High-Capacity cDNA Reverse Transcription Kit and the TaqMan PreAmp Master Mix (Applied Biosystems). The resulting cDNA was then amplified using *β-actin* and *cecr1b* E2Fwd-E4Rev (Ex3 skipping) primers (Supplementary Table 2). The PCR bands were isolated from a 2% agarose gel, purified, and subjected to sequencing (Wizard® SV Gel and PCR Clean-Up System (Promega). The *tnfα*-ATG morpholino (*tnfα*-ATGMO) was injected at a dose of 0.125-pmol/embryo.

For CRISPR-Cas9 genome editing, the web tool crispor.tefor.net was used to design two different 20-base pair long crRNAs specific to *S. pyogenes* Cas9 (SpCas9)^87^. The zebrafish GRCz11 genome assembly served as the reference for designing the two crRNAs for each gene (Supplementary Table 3). Synthetic sgRNAs were synthesized by IDT as Alt-R CRISPR-Cas9 crRNA (2 nmol/ml) and Alt-R CRISPR-Cas9 tracrRNA (5 nmol/ml). A 100 μM stock solution of the two crRNAs and tracrRNA was prepared using IDT Nuclease-Free Duplex Buffer. For injection, 1 μl of each crRNA stock solution was combined with 1 μl of tracrRNA stock solution. The mixture was then incubated at 95 °C for 5 min, cooled to room temperature, and supplemented with 0.2 μl of 10X CRISPR buffer (20 mM Hepes-NaOH pH 7.5, 0.15 M KCl). The injection mix was prepared with 0.5 μl of each crRNA/tracrRNA solution and 1 μl of Alt R S.p. Cas9 nuclease V3 (10 μg/μl) protein (IDT). Subsequently, 3 μl of the injection mix comprising the two crRNA (*cecr1b*-crRNA1+*cecr1b*-crRNA2) and tractRNA were micro-injected into one-cell stage zebrafish embryos. To validate genome editing, PCR was performed. Genomic DNA was extracted from pools of 16 randomly selected 24 hpf embryos using proteinase K digestion (0.17 mg/mL, Roche Diagnostics) in lysis buffer (10 mM Tris, pH 8.0, 10 mM NaCl, 10 mM EDTA). The mixture was incubated at 55 °C for 1 h and 30 min, followed by inactivation at 95 °C for 15 min. Each target genomic locus was PCR-amplified using Phusion High-Fidelity DNA polymerase (ThermoFisher Scientific, # F-530XL) and analyzed on a 1% agarose gel to detect the presence of an additional, aberrant band indicating successful genome editing (refer to Supplementary Table 2 for primer sequences).

### T7 Endonuclease I Assay

The T7 endonuclease I assay employs a mismatch-specific DNA endonuclease (T7E1) to identify the mutations in a target DNA region. This process involves PCR amplification of the targeted region, followed by digestion with T7E1. To reveal that the PCR amplicons obtained from *cecr1b*-LoF (injected with the two guides simultaneously) contained a series of small indels not visible by simple PCR amplification, we subjected the PCR product to T7 assay with the EnGen® Mutation Detection Kit (New England Biolabs, Ipswich, UK). The presence of digested PCR fragments of various dimensions was tested by agarose gel electrophoresis.

### Fluorescence activated cell sorting (FACS) analysis

Embryos dissociation was obtained^88^. GFP+ cells were sorted from Tg(*fli1a:*GFP)^*y1*^ ^63^ embryos at 32-34 hpf using a BD FACSMelody Cell Sorter (BD Bioscience, Franklin Lakes, NJ, USA). Cells were separated from debris using forward scatter (FSC-A) and side scatter area (SSC-A). Singlet gating then excluded doublet cells by analyzing side scatter width (SSC-W) with side scatter height (SSC-H). Further refinement was done on the dot plot (cut-P2), by examining FSC-W against FSC-H in the P3 gate population. Endothelial cells, identified by green fluorescent protein (GFP+), were selected from the cell populations. A 488 nm laser line was used for fluorescence detection. The gating strategy table provided details on the number of cells per gate (#Events), the percentage of cells in the parent gate (%Parent), and the total percentage of cells (%Total).To ensure accuracy in fluorescence detection and to eliminate the interference of auto-fluorescence, embryos of the wild-type AB strain were used to set the gate and exclude auto-fluorescence of cells.

### Time-lapse confocal microscopy

Time-lapse experiments were performed utilizing a Yokogawa spinning Disk (ECLIPSE Ti2-E) confocal microscope with W1-SoRa module (Nikon). 30 hpf embryos were anesthetized in 0.016% tricaine solution and included laterally in 1% low-melting point agarose in 35 mm confocal Petri dishes to provide stability during imaging. Included embryos were soaked in 0.016% tricaine solution to prevent the gel from drying out. Embryos were acquired for 6 h every 5 min (magnification 20X, step size 0.5μm). Images were processed and analyzed using the NIS Element Analysis Software (Nikon) and the Fiji (ImageJ) software.

### Real-time qPCR analyses

For real-time quantitative-PCR (RT-qPCR) analyses, total RNA was extracted from the embryos with NucleoZOL (Macherey Nagel) following the manufacturer’s instructions. For each sample, 1 μg of RNA was treated with DNAseI RNase-free (Roche Diagnostics, Basel, Switzerland) to avoid genomic contamination and retro-transcribed to cDNA using GoScript Reverse Transcription system (Promega, Madison, Wisconsin USA). qPCR reactions were performed using the iQ SYBR Green Super Mix (Promega) and the 384-well QuantStudio™ 5 Real-Time PCR System (Applied Biosystem, Whaltam, MA, USA). Expression levels of each analyzed gene were normalized to the zebrafish *rpl8* housekeeping gene and estimated with the 2 − ΔΔCt comparative method. Primers used for the analyses are listed in Supplementary Table 2.

### Neutrophil staining and count

Mature neutrophils were stained using the Leucognost-POX colorimetric assay^56^. Embryos were fixed in 4% paraformaldehyde (PFA) solution (Sigma-Aldrich) in phosphate buffer saline (PBS) for 2 h at room temperature (RT). Subsequently, they were washed in PBS and transferred to a staining solution for 10–15 min under continuous monitoring. After staining, the embryos were washed in PBS. Images of each stained embryo were acquired using a stereomicroscope equipped with a digital camera and LAS Leica imaging software (Leica, Wetzlar, Germany). The acquired images were processed and analyzed using Fiji (ImageJ) software. For the analysis, a region of interest (ROI) was defined around the CHT, and the mature neutrophils within this ROI were manually counted.

### Whole mount in situ hybridization (WISH) and quantifications

Whole-mount in situ hybridization (WISH) experiments were conducted according to a standardized protocol optimized for each specific probe used^89^. Each stained embryo was captured using a stereomicroscope equipped with a digital camera and LAS Leica imaging software (Leica, Wetzlar, Germany). The acquired images were processed and analyzed using Fiji (ImageJ) software. The signal intensity and area were quantified^90^.

### Chemical treatments

To prepare for pharmacological treatments, 10 mM stock solutions of CGS15943 (Tocris, BioTechne) and 50 mM stock solutions of NECA (Merck Millipore) were dissolved in dimethyl sulfoxide (DMSO). Additionally, 25 mM stock solution of H89 dihydrochloride (Tocris, BioTechne) was prepared in distilled water. Embryos were treated in 6-well plates with a maximum of 30 embryos/well using E3 medium supplemented with PTU. The treatment durations varied based on the experimental design, as described in the Results section. Treatments were initiated at 5–8 somites stage to 34–36 hfp, from 50% epiboly to 26–32 hpf, or from 24 hpf and 36 hpf to 3 dpf. CGS15943, NECA, and H89 dihydrochloride were administrated to a final concentration of 10, 20, and 10 μM, respectively; for the negative control group, the equivalent DMSO volume was administrated to untreated embryos.

### rhADA2 injection

The recombinant human ADA2 protein (rhADA2) (Recombinant Human Adenosine Deaminase 2/CECR1 Protein, CF, R&D) was diluted in sterile PBS at various concentrations (1:100, 1:75 and 1:50) immediately before injection. Intravascular injection of 1 nl of the rhADA2 dilution was performed into the duct of Cuvier of previously anesthetized 26–28 hpf larvae so that 17, 23, or 35 pg of the rhADA2 were injected in each embryo. As a negative control, a corresponding dilution of the rhADA2 resuspension vehicle (20 mM HEPES, 200 mM NaCl, 20% glycerol) was injected into control and *cecr1b*-LoF embryos.

### Immunofluorescence and apoptosis stainings

HSPC proliferation rate was assessed by double immunofluorescence staining for GFP and phoshohistone-H3 (p-H3) in Tg(CD41:GFP) embryos. Embryos were fixed in 4% PFA in PBS for 2 h at RT and rinsed several times in PBS and washing buffer (PBS 1X, Triton X100 1%, DMSO 0.2%). After incubation in blocking buffer (PBS1X, Triton X 0.1%, DMSO 1%, sheep serum 5%) for 2 h at RT and the following primary antibodies were added for overnight incubation at 4 °C: mouse anti-GFP (dilution 1:2000; Sigma-Aldrich, catalog number: MAB3580, lot number: 3729365) and rabbit anti-p-H3 (dilution 1:200; Sigma-Aldrich, catalog number: MFCD01633984). The following secondary antibodies were added to the blocking buffer: Alexa Fluor 488 conjugated goat-anti-mouse antibody (1:1000) and Alexa Fluor 546 conjugated goat-anti-rabbit antibody (1:200) (Invitrogen Life Technologies, Ref number A11001, Lot number 2090562 and Ref number A11010, Lot number 1971417, respectively).

Fluorescence imaging was performed using confocal microscopy. To examine the proliferation rates, the number of double positive cells (GFP+/p-H3+) was counted and normalized against the total GFP+ cells in the CHT region. For the detection of apoptotic cells in Tg(CD41:GFP) fixed larvae, the Click-iT Plus TUNEL assay was used with the Alexa Fluor 647 picolyl azide dye, following the manufacturer’s instruction (Invitrogen Life Technologies). The fluorescence images were acquired using a Leica, TCS-SPII confocal microscope following the inclusion of the embryos in 1% low melting point agarose gel in 35 mm confocal Petri dishes to provide stability during imaging. Images were processed and analyzed using Fiji (ImageJ) software.

### In-vivo analyses with transgenic lines

Analyses with the Tg(CD41:GFP)^71^, Tg(*tnfα*:GFP)^57^; Tg(*kdrl*:GFP)^70^ and Tg(*runx1*:citrine);Tg(*kdrl*:mCherry)^69^ transgenic lines were performed with an epifluorescence stereomicroscope (MZFLIII, Leica, Wetzlar, Germany) equipped with a DFC 480-R2 digital camera digital camera.

### Statistics and reproducibility

Data were presented as means ± standard deviation (SD) or standard error of the mean (SEM), as indicated in the figure captions. To compare different experimental groups, the t-student test, Chi-square test, and One-way ANOVA (with Tukey’s correction) were employed. Statistical significance was determined by p-values, where values less than 0.05 (*), 0.01 (**), and 0.001 (***) were considered statistically significant. All analyses were conducted with the software package GraphPad Prism 9.0.0 (GraphPad, San Diego, CA). All experiments were conducted with a minimum of three biologically independent replicates.

### Supplementary information

Supplementary material

Description of Additional Supplementary Files

Supplementary Movie 1

Supplementary Movie 2

## Data Availability

All raw data related to the manuscript are currently collected in the dataset named “Replication data for DADA2 project”, fully available in the Dataverse Repository at the following link: 10.13130/RD_UNIMI/WM3DGM^91^. The dataset contains raw data of this project which are organized in a set of folders, each one corresponding to a figure. Each folder is then organized in subfolders, each one containing raw data, graphpad/excel files containing numerical measurements, and a “README” file describing the content of each folder.
