# Supplementary material for: ADA2 regulates inflammation and hematopoietic stem cell emergence via the A2bR pathway in zebrafish
Source: Commun Biol. 2024 May 22;7:615. doi: 10.1038/s42003-024-06286-3 (PMC11111730; doi:10.1038/s42003-024-06286-3)
Supplement: Supplementary file 1 — Supplementary material [file 42003_2024_6286_MOESM1_ESM.pdf]

ADA2 regulates inflammation and hematopoietic stem cell emergence via the A2bR pathway in zebrafish

Alessia Brix[1], Laura Belleri[1,2], Alex Pezzotta[1], Emanuela Pettinato[3], Mara Mazzola[1], Matteo Zoccolillo[3], Anna Marozzi[1], Rui Monteiro[4], Filippo Del Bene[2], Alessandra Mortellaro[3*], Anna Pistocchi[1*]

[1] *Department of Medical Biotechnology and Translational Medicine, Università degli Studi di Milano, L.I.T.A. via Fratelli Cervi 93 20054, Segrate (Milan), Italy*

[2] *Institut de la Vision, Department of Development 17, rue Moreau 75012, Paris, France*

[3] *San Raffaele Telethon Institute for Gene Therapy (SR-Tiget), IRCCS San Raffaele Scientific Institute, via Olgettina 60, 20132 Milan, Italy*

[4] *Institute of Cancer and Genomic Sciences, University of Birmingham, Birmingham, Edgbaston B15 2TTB, United Kingdom*

* These authors contributed equally

Correspondence to:

Anna Pistocchi, Department of Medical Biotechnology and Translational Medicine, Università degli Studi di Milano, L.I.T.A. Via Fratelli Cervi 93, 20054, Segrate, Italy. Tel. +39 02 50330466 anna.pistocchi@unimi.it

Alessandra Mortellaro, San Raffaele Telethon Institute for Gene Therapy (SR-Tiget), IRCCS San Raffaele Scientific Institute, Via Olgettina 60, 20132 Milan, Italy. Tel. +39 02 26435326
mortellaro.alessandra@hsr.it

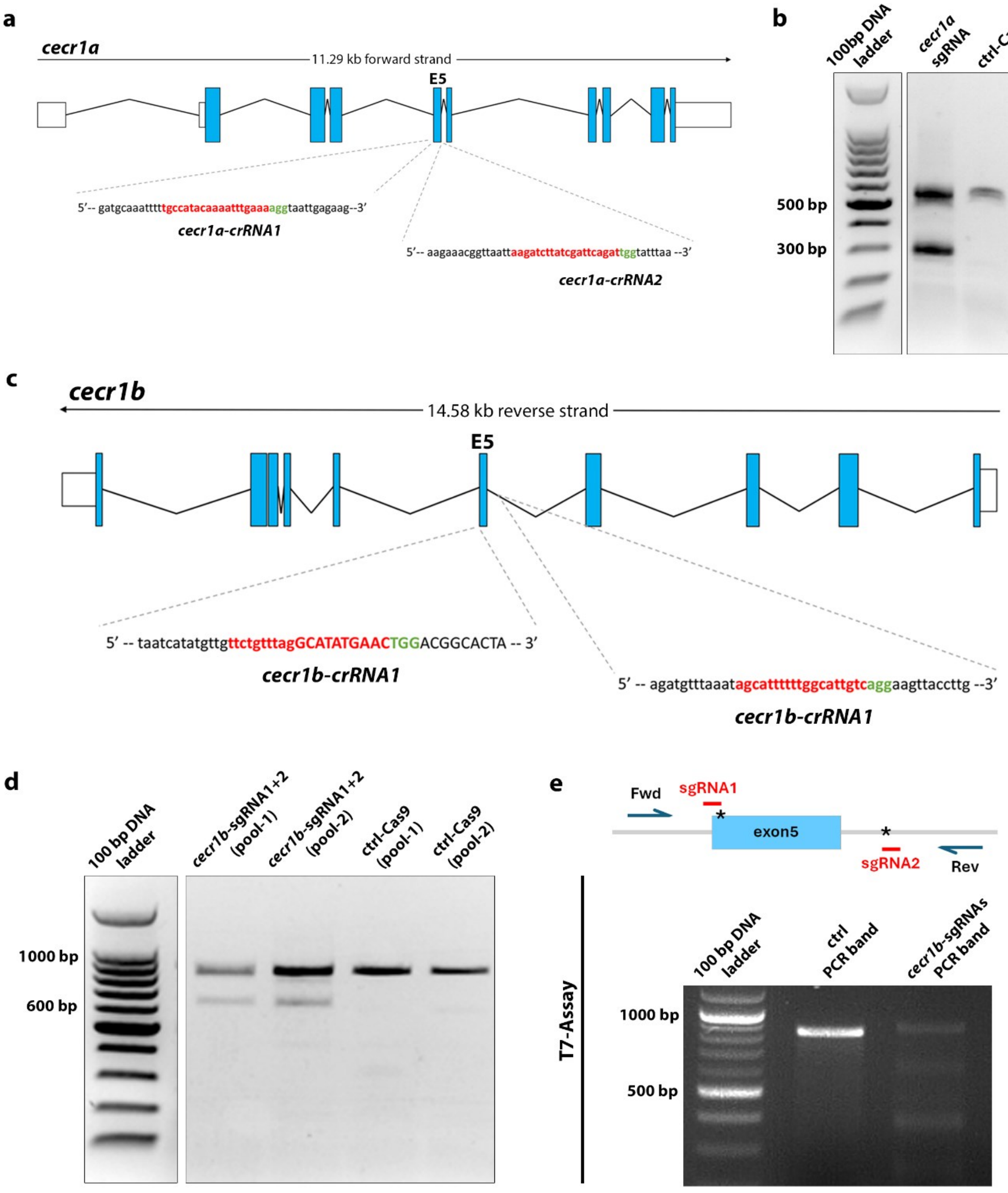

Supplementary Figure 1. Molecular validation of *cecr1a*- and *cecr1b*-sgRNA zebrafish models. **(a)** Schematic representation of the *cecr1a* genomic region targeted in the exon 5 by the two different *cecr1a* crRNAs 1 and 2(*cecr1a*-sgRNA). crRNA and PAM sequences are highlighted in red and green, respectively. Lowercase = intronic region. **(b)** Agarose gel electrophoresis of the amplification products obtained with *cecr1a* primers flanking the genomic region cut by the *cecr1a*-sgRNAs in ctrl Cas9 and *cecr1a*-sgRNAs injected embryos. (**c**) Schematic representation of the *cecr1b* genomic region targeted at the level of exon 5 by the two different indicated *cecr1b* crRNAs 1 and 2 (*cecr1b*-sgRNA) sequence and PAM sequence are highlighted in red and green, respectively. Lowercase = intronic region, uppercase = exonic region. **(d)** Agarose gel electrophoresis of the amplification products obtained with *cecr1b* primers flanking the genomic region cut by the *cecr1b*-sgRNAs in ctrl Cas9 and *cecr1a*-sgRNAs

injected embryos belonging to two different pools. **(e)** Schematic representation of the PCR strategy utilized and results of the T7 endonuclease I assay revealing multiple bands obtained after T7 digestion and due to indels contained in the PCR amplification products of *cecr1b*-sgRNA1+2 injected embryos. Exon 5 is represented as a blue box, while the surrounding intronic regions are represented as grey lines. Forward (Fwd) and reverse (Rev) primers are represented as arrows, while the two *cecr1b*-sgRNAs are indicated as red lines. Asterisks indicate where the sgRNAs-mediated DNA cleavages occur.

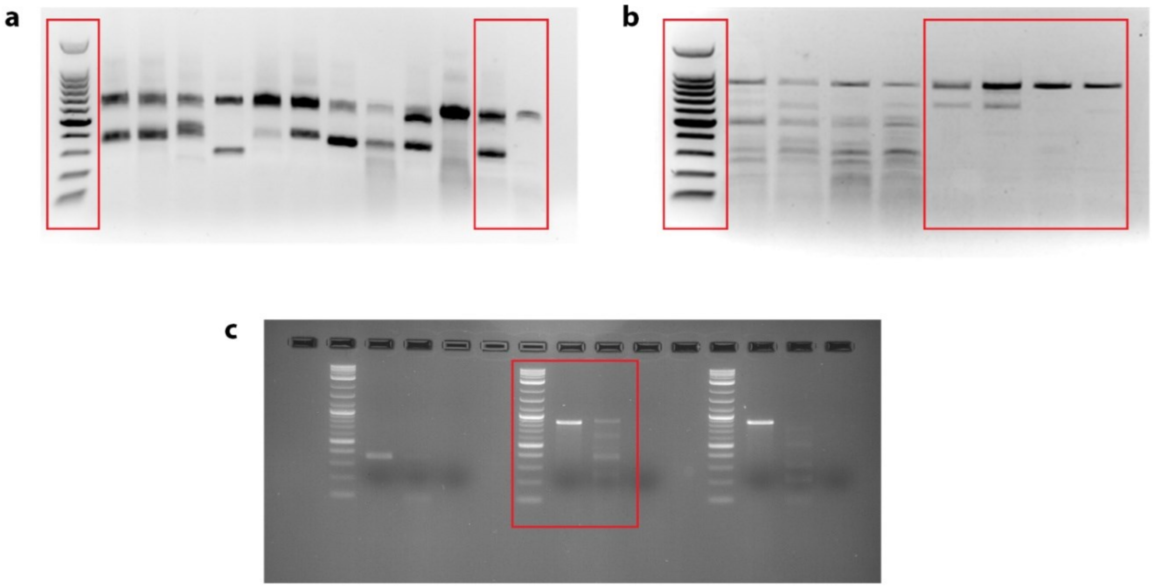

Supplementary Figure 2. **Original, uncropped and unedited agarose gel images utilized for the molecular validations of the *cecr1b*-LoF zebrafish model. (a)**, **(b)** and **(c)** correspond to the Supplementary Figure 1b, Supplementary Figure 1d and Supplementary Figure 1e, respectively. Red rectangles indicate the parts of the gels reported in Supplementary Figure 1.

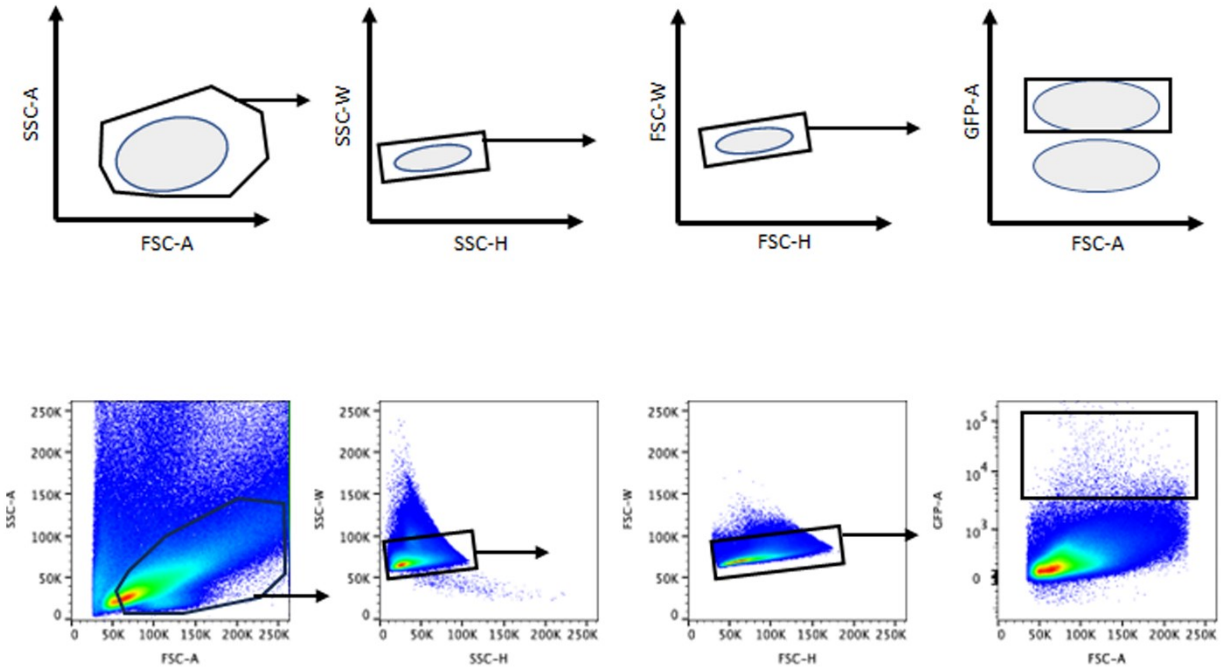

Supplementary Figure 3. Gating Strategy for FACS sorting of *fli1a*-GFP+ endothelial cells from Tg(*fli1a*:GFP)^{y1} embryos. Cells were separated from debris using forward scatter (FSC-A) and side scatter area (SSC-A). Singlet gating then excluded doublet cells by analyzing side scatter width (SSC-W) with side scatter height (SSC-H). Further refinement was done on the dot plot (cut-P2), by examining FSC-W against FSC-H in the P3 gate population. Endothelial cells, identified by green fluorescent protein (GFP+), were selected from the cell populations. A 488 nm laser line was used for fluorescence detection.

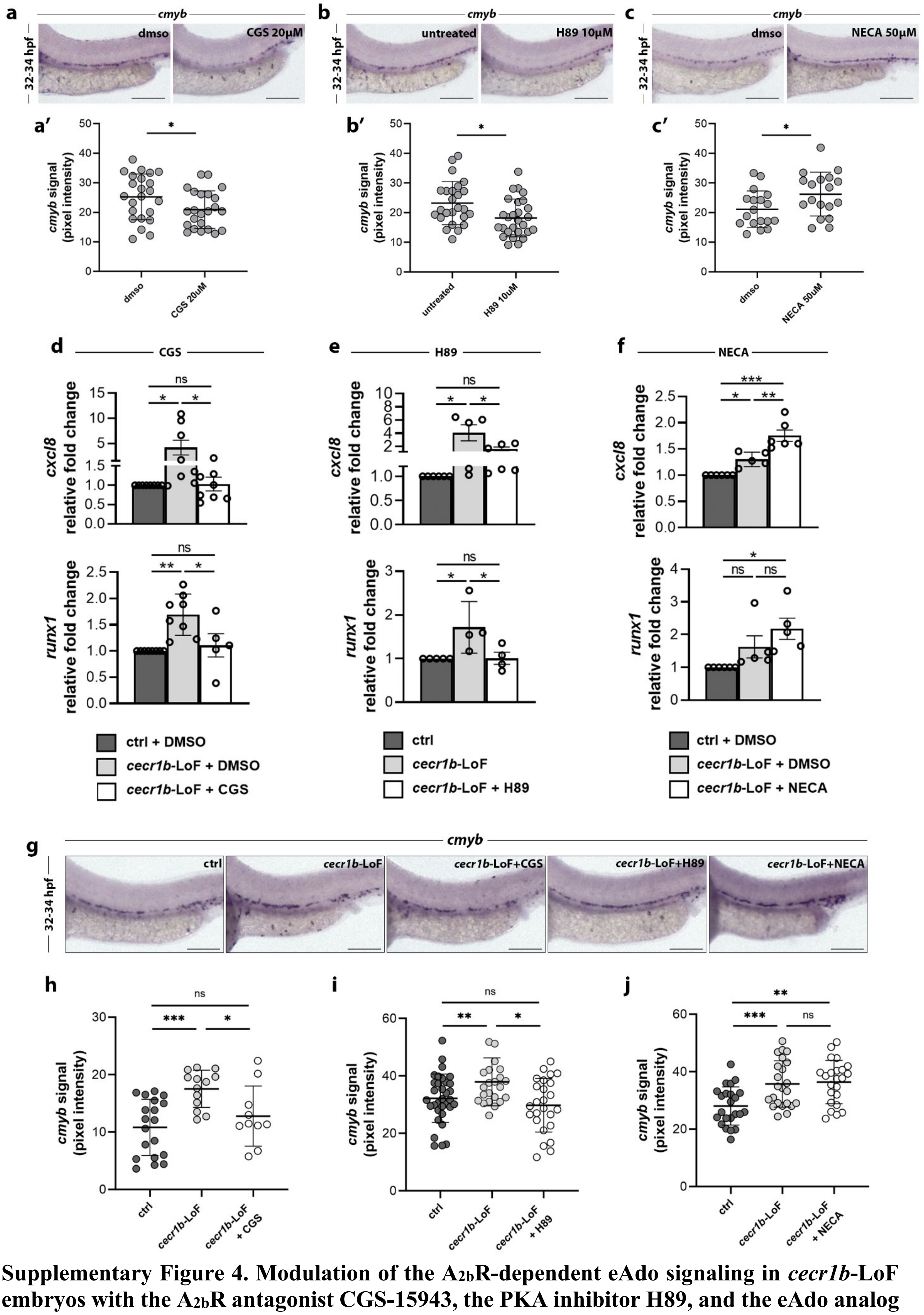

Supplementary Figure 4. Modulation of the A$_{2b}$R-dependent eAdo signaling in *cecr1b*-LoF embryos with the A$_{2b}$R antagonist CGS-15943, the PKA inhibitor H89, and the eAdo analog NECA. (a-c) Representative WISH images of the *cmyb* signal in the trunk region of 32-34 hpf

wild-type embryos treated with 20 μM CGS **(a)**, 10 μM H89 **(b),** and 50 μM NECA **(b)**. Scale bar: 100 microns. **(a'-c')** Quantification of *cmyb* signal intensity in the HE of wild-type embryos following CGS, H89, and NECA administration. Each dot represents the count of a single embryo. Data are presented with mean±SD. Statistical significance was assessed by a two-tailed, unpaired t-student test. *$p < 0.05$ (CGS treatment: DMSO, n=23 embryos; CGS 20μM, n=23 embryos. H89 treatment: untreated, n=25; H89 embryos 10μM, n=27 embryos. NECA treatment: DMSO, n=18 embryos; NECA 50μM, n=18 embryos). (**d-f**) RT-qPCR analyses of the A_{2b}r downstream genes *cxcl8* and *runx1* in 32-34 hpf control embryos, *cecr1b*-LoF embryos and *cecr1b*-LoF embryos treated with CGS (**d**), H89 (**e**), and NECA (**f**). Data are presented as mean±SEM. Statistical significance was assessed by Ordinary one-way ANOVA test with Tukey's correction; ***$p<0.001$; **$p<0.002$; *$p<0.05$ ns, not significant. **(g)** Representative WISH images of the trunk region of 32-34 hpf control embryos and *cecr1b*-LoF embryos untreated or treated with CGS, H89 and NECA, stained for the HSPCs marker *cmyb*. Scale bar: 100 microns. **(h-j)** Quantification of *cmyb* signal intensity in the HE of *cecr1b*-LoF embryos of the different experimental categories. Statistical significance was assessed by Ordinary one-way ANOVA test with Tukey's correction; ***, $p<0.001$; **, $p<0.002$; *, $p < 0.05$; ns, not significant (CGS treatment: ctrl, n=19 embryos; *cecr1b*-LoF, n =13 embryos; *cecr1b*-LoF+CGS, n=10 embryos. H89 treatment: ctrl, n=33 embryos; *cecr1b*-LoF, n =23 embryos; *cecr1b*-LoF+H89, n=25 embryos; n=27 embryos. NECA treatment: ctrl, n=23 embryos; *cecr1b*-LoF, n =23 embryos; *cecr1b*-LoF+NECA, n=23 embryos).

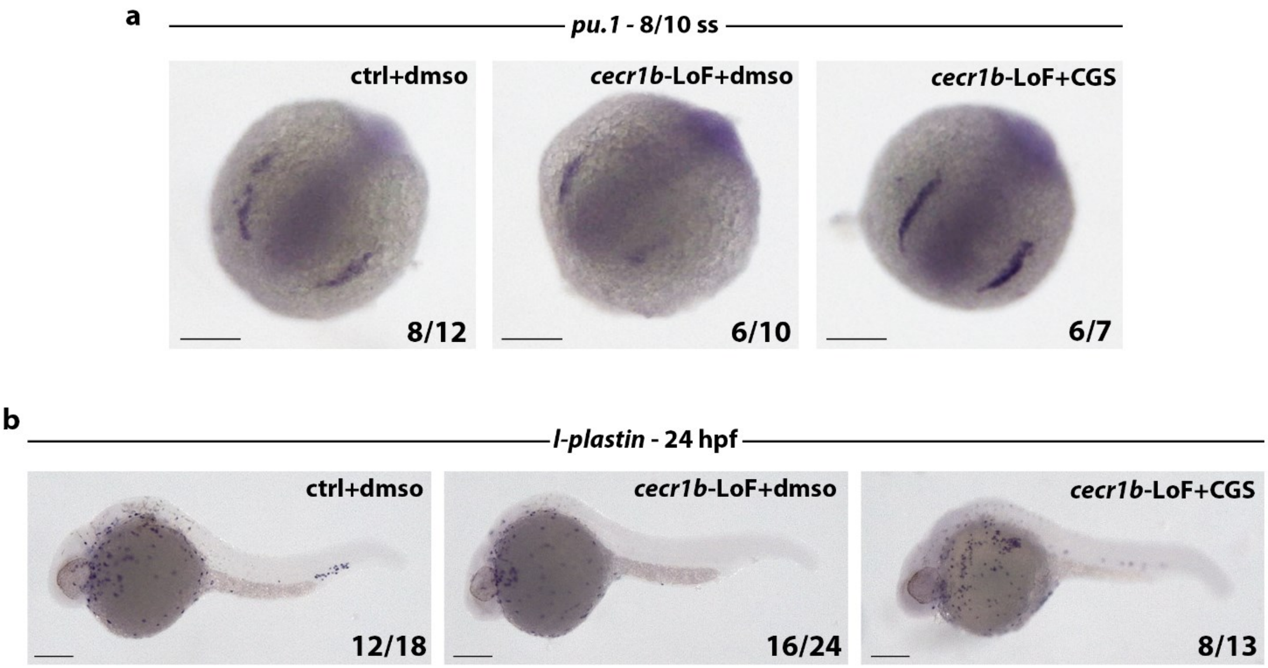

Supplementary Figure 5. **Analysis of primitive myeloid cells in *cecr1b*-LoF embryos and correction through A2r pathway inhibition.** (**a**) Representative WISH images of 8-10 ss embryos stained with the myeloid precursors marker *pu.1*, dorsal view. Scalebar = 300 microns. Numbers indicate the embryos belonging to the representative phenotype of each category, shown in the image. (**b**) Representative WISH images of 24 hpf embryos labeled with the leukocyte marker *l-plastin*. Scalebar = 300 microns. Numbers indicate the embryos belonging to the representative phenotype of each category, shown in the image.

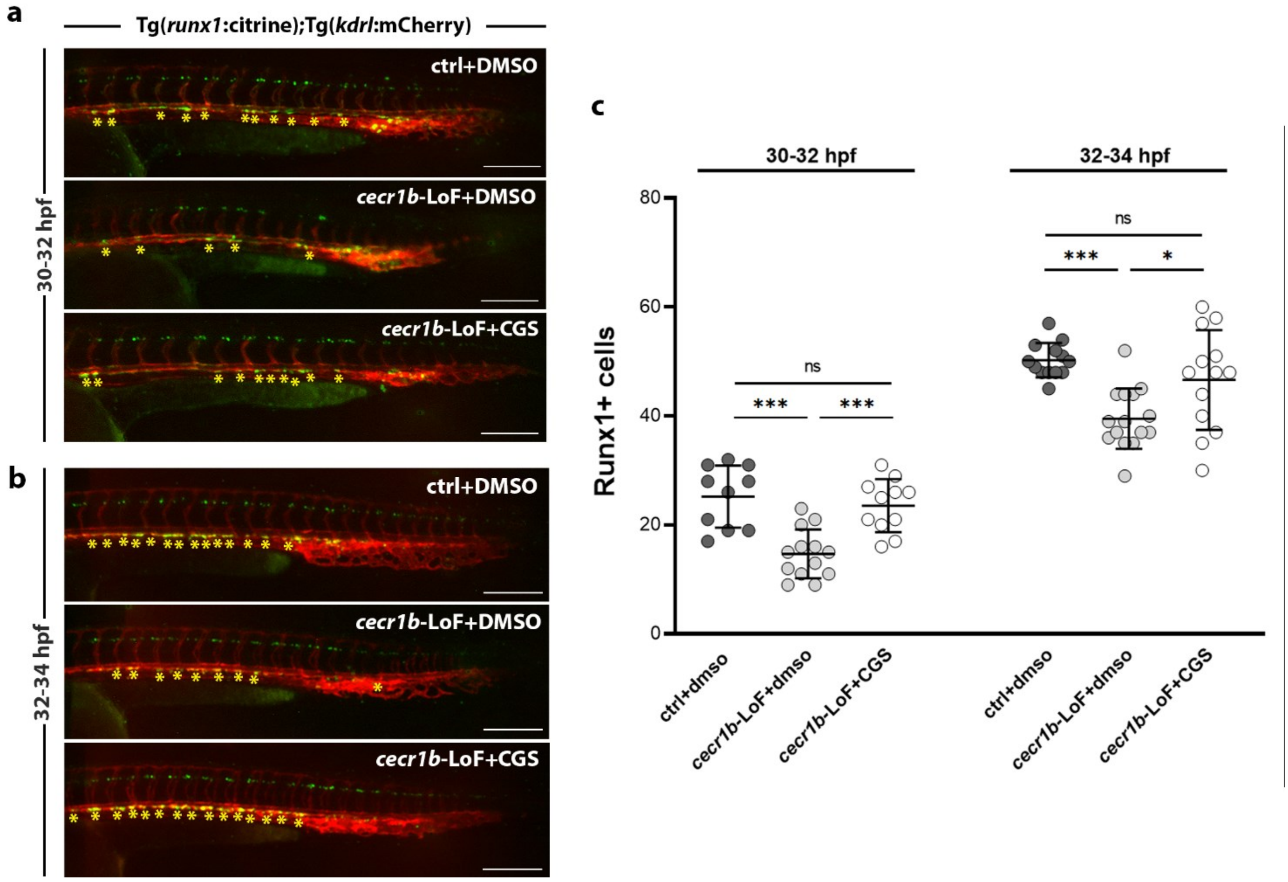

Supplementary Figure 6. Runx1 expression in *cecr1b*-LoF embryos in the presence or in the absence of $A_{2b}R$ pathway modulation. (a,b) Representative fluorescence images of the trunk-tail region of Tg(*runx1*:citrine);Tg(*kdrl*:mCherry) embryos of the different experimental conditions at 30-32 hpf and 32-34 hpf. Specifying Runx1+ HSPCs are indicated by yellow asterisks. Scale bar: 150 µm. (**c**) Quantification graph of Runx1+ HSPCs at the indicated time points. Each dot represents the count of a single embryo. Data are presented as mean± SD. Statistical significance was assessed by Ordinary one-way ANOVA test with Tukey's correction; ***$p<0.001$; *$p < 0.05$; ns: not significant (30-32 hpf: ctrl, n=10 embryos; *cecr1b*-LoF, n=13 embryos; *cecr1b*-LoF+CGS, n=11 embryos. 32-34 hpf: ctrl, n=13 embryos; *cecr1b*-LoF, n=15; *cecr1b*-LoF+CGS, n=11 embryos).

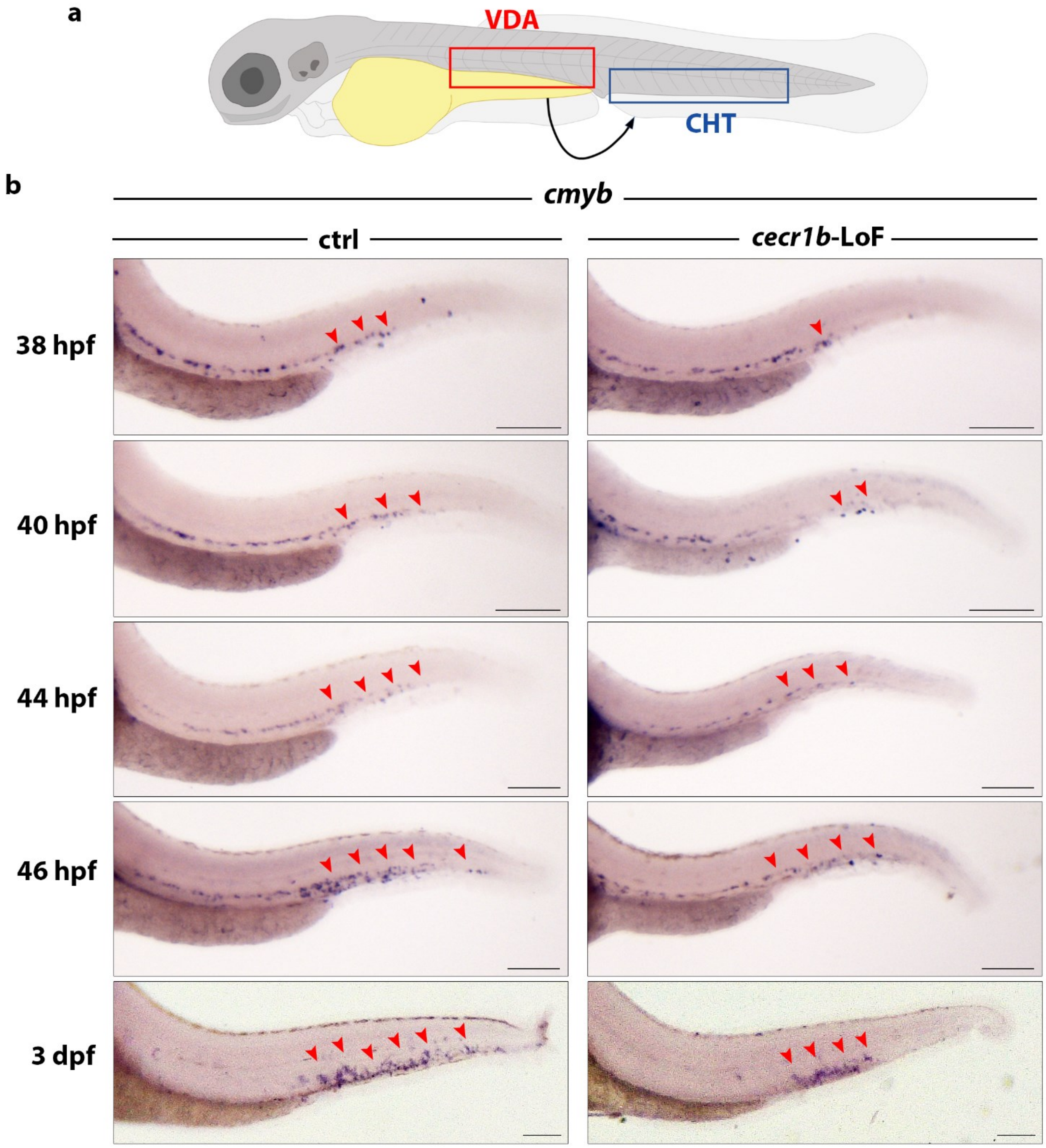

Supplementary Figure 7. Analyses of HSPCs migration toward the caudal hematopoietic tissue in controls and *cecr1b*-LoF embryos at different developmental stages. (A) Schematic representation depicts a 3 dpf embryo highlighting the VDA, where HSPCs undergo specification, and the CHT, which serves as the migration site for these specialized cells. **(B)** Representative images of time-point WISH analyses for the *cmyb* marker in control and *cecr1b*-LoF embryos spanning from 38 hpf to 3 dpf. Red arrowheads denote *cmyb*+ cells colonizing the CHT. Scale bar: 150 microns.

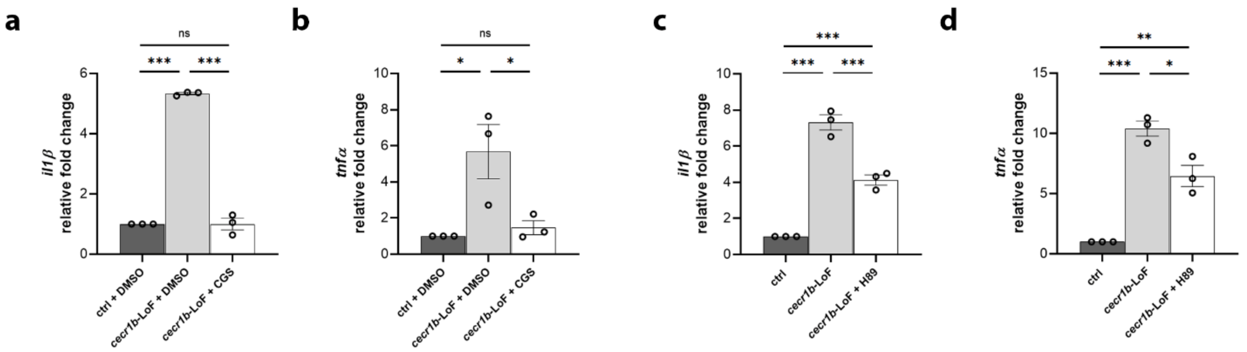

Supplementary Figure 8. *tnfα* modulation rescue HSPCs defects of *cecr1b*-LoF embryos. (A-D) RT-qPCR analyses for the pro-inflammatory cytokines **(A-B)** *il1β* and *tnfα* in 32-34 hpf embryos treated with **(A-B)** CGS and **(C-D)** H89. Data are presented as mean±SEM, and statistical significance was assessed by Ordinary one-way ANOVA test with Tukey's correction; ***p<0.001; **p<0.002; *p < 0.05; ns, not significant.

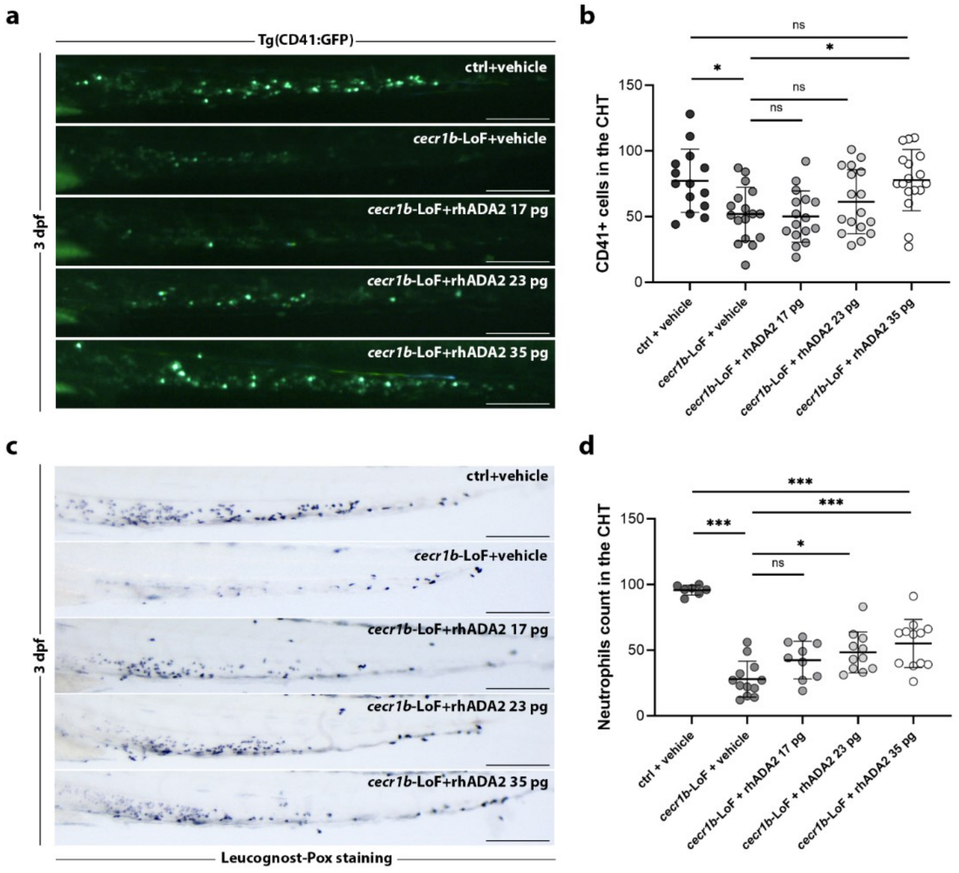

Supplementary Figure 9. Dose-dependent ADA2 supplementation improves HSPC number and CHT colonization in *cecr1b*-LoF embryos. (a, b) Representative fluorescence images of the CHT region of 3 dpf Tg(CD41:GFP) embryos and quantification of the CD41+ HSPCs cells in controls, *cecr1b*-LoF, and *cecr1b*-LoF embryos administrated with different doses of the rhADA2. Scale bar: 300 microns. Statistical significance was assessed by Ordinary one-way ANOVA test with Tukey's

correction; ***p<0.001; **p<0.002; *p < 0.05; ns, not significant. **(c, d)** Representative bright-field images and quantification of neutrophils stained with the Leucognost-Pox colorimetric assay in the CHT of 3 dpf embryos. Scale bar: 300 microns. Statistical significance was assessed by Ordinary one-way ANOVA test with Tukey's correction; ***p<0.001; **p<0.002; *p < 0.05; ns, not significant (ctrl+vehicle, n=14 embryos; *cecr1b*-LoF+vehicle, n=17 embryos; *cecr1b*-LoF+rhADA2-17pg, n=16 embryos; *cecr1b*-LoF+rhADA2-23pg, n=17 embryos; *cecr1b*-LoF+rhADA2-35pg, n=17 embryos).

Movie 1. HSPCs emerging from the hemogenic endothelium of a Tg(*kdrl*:GFP) control embryo recorded from 30 to 36 hpf. Magnification of an area corresponding to 3-somites of the trunk region.

Movie 2. HSPCs emerging from the hemogenic endothelium of a Tg(*kdrl*:GFP) *cecr1b*-LoF embryo recorded from 30 to 36 hpf. of an area corresponding to 3-somites of the trunk region.

SUPPLEMENTARY TABLES:

Supplementary Table 1. Morpholino sequences

morpholino name	Sequence (5'-3')
cecr1b-ATGMO	GCTTATGCTACTCATTGCTCCCAGC
cecr1b-sMO	TAACTTTGATGTTCGCTCACCTGTT
tnfα-ATGMO	AGCTTCATAATTGCTGTATGTCTTA

Supplementary Table 2. Primers list

primer name	Sequence (5'-3')	Purpose
β-actin Fwd	TGTTTTCCCCTCCATTGTTG	RT-PCR for cecr1b-sMO validation

β-actin Rev	TTCTCCTTGATGTCACGGAC	RT-PCR for cecr1b-sMO validation
cecr1b-E2Fwd	CAAAGTGCGGCACATCATACA	RT-PCR for cecr1b-sMO validation
cecr1b-E4Rev	TGTGATCAGCCCAGACAGAG	RT-PCR for cecr1b-sMO validation
cecr1a-Fwd	GCAACTCAAATCTGTCAGAGGAA	PCR for cecr1a-sgRNA validation
cecr1a-Rev	AGCCGCACTCTGAGGAAAAAT	PCR for cecr1a-sgRNA validation
cecr1b-Fwd	GAGATGATTCATCTTAAGGTGC	PCR for cecr1b-sgRNA validation
cecr1b-Rev	CCTGTTAGACTAGAAGTGCC	PCR for cecr1b-sgRNA validation
rpl8-Fwd	CTCCGTCTTCAAAGCCAATG	RT-qPCR
rpl8-Rev	TCCTTCACGATCCCCTTGAT	RT-qPCR
cxcl8-Fwd	CGACGCATTGGAAAACACAT	RT-qPCR
cxcl8-Rev	TGTCATCAAGGTGGCAATGA	RT-qPCR
runx1-Fwd	CTCTGAGCAGTTGAGGCGAA	RT-qPCR
runx1-Rev	CTGCCGGGAGTCGGGAAT	RT-qPCR
cmyb-Fwd	GACACAAAGCTGCCCAGTTG	RT-qPCR
cmyb-Rev	GCTCTTCCGTCTTCCCACAA	RT-qPCR
il1β-Fwd	TGGACTTCGCAGCACAAAATG	RT-qPCR
il1β-Rev	CGTTCACTTCACGCTCTTGGATG	RT-qPCR
tnfα-Fwd	CTTCACGCTCCATAAGACCC	RT-qPCR
tnfα-Rev	GCCTTGGAAGTGAAATTGCC	RT-qPCR

Supplementary Table 3. crRNA sequences

crRNA name	Sequence (5’-3’)
cecr1a-crRNA1	GATGCAAATTTTTGCCATACAAAATTTGAAAAGGTAATTGAGAAG
cecr1a-crRNA2	AAGAAACGGTTAATTAAGATCTTATCGATTCAGATTGGTATTTAA
cecr1b-crRNA1	TAATCATATGTTGTTCTGTTTAGGCATATGAACTGGACGGCACTA
cecr1b-crRNA2	AGATGTTTAAATAGCATTTTTTGGCATTGTCAGGAAGTTACCTT
