# Supplementary material for: ADA2 regulates inflammation and hematopoietic stem cell emergence via the A2bR pathway in zebrafish
Source: Commun Biol. 2024 May 22;7:615. doi: 10.1038/s42003-024-06286-3 (PMC11111730; doi:10.1038/s42003-024-06286-3)
Supplement: Supplementary file 2 — Description of Additional Supplementary Files [file 42003_2024_6286_MOESM2_ESM.pdf]

Description of Additional Supplementary Files

File name: Supplementary Movie 1.

Title: HSPCs budding from the HE.

Description: HSPCs emerging from the hemogenic endothelium of a Tg(kdrl:GFP) control embryo recorded from 30 to 36 hpf. Magnification of an area corresponding to 3- somites of the trunk region.

File name: Supplementary Movie 2.

Title: defective budding of cecr1b-deficient HSPCs from the HE

Description: HSPCs emerging from the hemogenic endothelium of a Tg(kdrl:GFP) cecr1b-LoF embryo recorded from 30 to 36 hpf. Magnification of an area corresponding to 3-somites of the trunk region.
